# Piezoresistive Cantilever Microprobe with Integrated Actuator for Contact Resonance Imaging

**DOI:** 10.3390/s25020332

**Published:** 2025-01-08

**Authors:** Tianran Ma, Michael Fahrbach, Erwin Peiner

**Affiliations:** 1 Institute of Semiconductor Technology (IHT), Technische Universität Braunschweig, Hans-Sommer-Straße 66, 38106 Braunschweig, Germany; 2 Laboratory for Emerging Nanometrology (LENA), Langer Kamp 6a/b, 38106 Braunschweig, Germany

**Keywords:** piezoelectric sensor, vibration model, numerical simulations, Wheatstone bridge, piezoresistivity

## Abstract

A novel piezoresistive cantilever microprobe (PCM) with an integrated electrothermal or piezoelectric actuator has been designed to replace current commercial PCMs, which require external actuators to perform contact-resonance imaging (CRI) of workpieces and avoid unwanted “forest of peaks” observed at large travel speed in the millimeter-per-second range. Initially, a PCM with integrated resistors for electrothermal actuation (ETA) was designed, built, and tested. Here, the ETA can be performed with a piezoresistive Wheatstone bridge, which converts mechanical strain into electrical signals by boron diffusion in order to simplify the production process. Moreover, a new substrate contact has been added in the new design for an AC voltage supply for the Wheatstone bridge to reduce parasitic signal influence via the EAM (Electromechanical Amplitude Modulation) in our homemade CRI system. Measurements on a bulk Al sample show the expected force dependence of the CR frequency. Meanwhile, fitting of the measured contact-resonance spectra was applied based on a Fano-type line shape to reveal the material-specific signature of a single harmonic resonator. However, noise is greatly increased with the bending mode and contact force increasing on viscoelastic samples. Then, to avoid unspecific peaks remaining in the spectra of soft samples, cantilevers with integrated piezoelectric actuators (PEAs) were designed. The numbers and positions of the actuators were optimized for specific CR vibration modes using analytical modeling of the cantilever bending based on the transfer-matrix method and Hertzian contact mechanics. To confirm the design of the PCM with a PEA, finite element analysis (FEA) of CR probing of a sample with a Young’s modulus of 10 GPa was performed. Close agreement was achieved by Fano-type line shape fitting of amplitude and phase of the first four vertical bending modes of the cantilever. As an important structure of the PCM with a PEA, the piezoresistive Wheatstone bridge had to have suitable doping parameters adapted to the boundary conditions of the manufacturing process of the newly designed PCM.

## 1. Introduction

In industrial manufacturing, monitoring of tools and workpieces is critical for quality assurance. Over the past decade, in alignment with the shift to Industry 4.0, characterized by the interconnection of machines and processes [1], sensors have been progressively included in production lines. A crucial metric in this context is the surface topography. It provides information about wear [2,3], manufacturing accuracy, surface texture, porosity, and cracks [4,5], which significantly influences the mechanical properties of microstructural surfaces [6]. In addition, the mechanical properties of the surface are crucial for numerous applications. For instance, stiffness is decisive for the properties of electromechanical nanogenerators [7,8,9,10,11,12]. The material inclusions, density, and residual stresses have a substantial influence on the function of additively manufactured components [5,13,14]. The mechanical properties of the surface indicate adhesion [15] and the state of annealing processes and other physical processes [16]. Various methods are used to measure topography and mechanical properties. These can be classified as non-contact and contact approaches. Non-contact approaches employ electrostatic, capacitive, acoustic, and optical methods [17,18], in which the optical methods are the most widely used. Optical methods have a lot of advantages, including speed, non-destructiveness, and high resolution. However, they are sensitive to severe absorption, reflection, and occlusion, only suitable for measuring translucent or opaque materials [19] but not for reflective or dark objects [17], and expensive and difficult to integrate into production equipment [18].

In contrast, contact methods, particularly nanoindentation and force–distance curves, have the benefit of directly investigating the mechanics of the surface, resulting in reproducible, accurate, and correct measurements [17]. Contact sensors measure in sequence. Thus, sample surfaces must be scanned to receive real information. This is known as SPM (Scanning Probe Microscopy). A description of several SPM types can be found in [20], which is divided into STM (Scanning Tunneling Microscopy) and AFM (Atomic Force Microscopy) by current or force measurement. However, they are severely limited by their measurement speed and scanned-area size. The measurement speed ranges from several seconds per indentation [21,22] to several minutes per indentation (maximum six indentations per second) [23] or one force–distance curve per ten seconds to 125 force–distance curves per second [24,25,26] (maximum 10,000 force–distance curves per second [27]). The scanned-area size is limited to 500 × 500 nm^2^ [28], 100 × 100 [29], 20 × 20 nm^2^ [30], and 2260 × 260 nm^2^ [31] for the high 3D resolution, etc.

Previously, a homemade CRI (Contact Resonance Imaging) setup [32,33] has been built, largely based on those from the field of AFM [31], to measure topography and mechanical properties in industrial environments with travel speeds up to 15 mms^−1^ [34]. Until now, measurements were performed using piezoresistive cantilever microprobes (PCMs) of type CAN50-2-5, manufactured by CiS Forschungsinstitut für Mikrosensorik GmbH, Erfurt, Germany [35,36]. However, these have some disadvantages. Since they were developed for topography tracing, external actuators required for contact resonance measurements have to be added. Mounted at the base of the PCM, they not only excite the beam into vibration but also its surroundings. As a result, the so-called “forest of peaks” [37,38] occurs when measuring viscoelastic samples in which the contact-resonance quality is greatly reduced. Here, the measurement signal contains further resonances that cannot be clearly assigned. Methods can remedy this, such as integrating the actuator into the cantilever, which shall only excite the beam into vibration. For this reason, sensors with integrated heating resistors in the cantilever as actuators have been designed [38], fabricated, and tested [39]. Another problem with the commercial sensors is that the integrated silicon probe tips wear out [40]. As a result, the contact radius is not constant during measurements, which leads to the drift of the contact resonance frequency [41]. To limit wear, probe tips can be coated with aluminum oxide and diamond-like carbon (DLC) [42]. However, this only provides a short-term improvement, as fractures of the probe tip during measurements expose the underlying silicon and leave it unprotected again. To guarantee reproducible measurements, future sensors are therefore equipped with wear-resistant solid diamond probe tips that are glued to the free end of the cantilever [42].

In this paper, we propose a piezoresistive cantilever with an integrated actuator and a glued conical solid diamond probe tip to address the above shortcomings of commercial sensors for CRI commercial sensors. Specifically, we introduce the design and optimization process of sensors with integrated electrothermal actuators (ETAs) or piezoelectric actuators (PEAs), finalize the production and testing of the electrothermal-actuated piezoresistive microprobe (ETA-PCM), and enhance the manufacturing process of the piezoelectric-actuated piezoresistive microprobe (PEA-PCM) accordingly.

## 2. Materials and Methods

**Figure 1:**
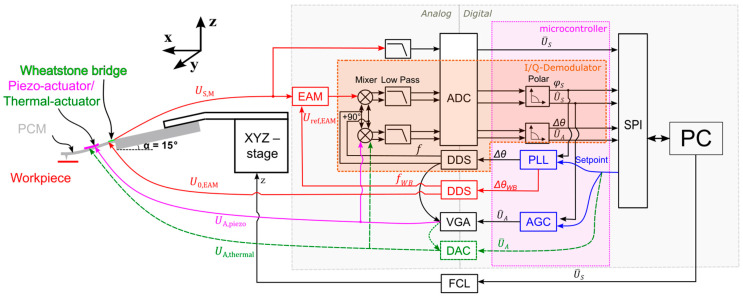
Contact-resonance imaging (CRI) system based on a piezoresistive cantilever microprobe (PCM) with an integrated actuator (electrothermal actuator, ETA or piezoelectric actuator, PEA). The homemade electronic system comprises an ADC (Analog-to-digital converter), two DDSs (Direct Digital Synthesizers), a VGA (Variable Gain Amplifier), a DAC (Digital-to-analog converter), an EAM (Electromechanical Amplitude Modulation), a PLL (Phase-Locked-Loop), an AGC (Automatic Gain Control), an FCL (Force Control Loop), an SPI (Serial Peripheral Interface), and a PC (Personal Computer).

Figure 1 shows the developed CRI system based on a PCM with an integrated ETA or PEA as part of a homemade electronic control and data acquisition system. The cantilever design is based on its dimensions on the CAN50-2-5, the dimensions of which are given in Table 1. The cantilever is actuated in vertical oscillations driven by an actuation signal $U_{A,thermal}\left(t\right)$ or $U_{A,piezo}\left(t\right)$, which are applied on the electrothermal or piezoelectric actuators, respectively:

(1) $$U_{A,thermal}\left(t\right)={\hat{U}}_{A}\times cos\left(2\pi ft\right)+{\bar{U}}_{A}$$

(2) $$U_{A,piezo}\left(t\right)={\hat{U}}_{A}\times cos\left(2\pi ft\right)$$

in which the actuation frequency f is given by a DDS (Direct Digital Synthesizer of $f=\frac{f_{clk^{∆\theta }}}{2^{B_{\theta (n)}}}$ with $f_{clk}$ as the system clock frequency, $B_{\theta (n)}$ as the phase width, and ∆θ as the phase increment value). The actuation amplitude ${\hat{U}}_{A}$ can be adjusted by controlling the gain of a VGA (Variable Gain Amplifier). For the electrothermal actuator, an additional DC component ${\bar{U}}_{A}$ of $U_{A,thermal}\left(t\right)$ is added by a DAC (Digital-to-analog converter), as shown by the green dotted line in Figure 1. The output signal from a piezoresistive Wheatstone bridge on the PCM:

(3) $$U_{S,M}\left(t\right)=U_{S}\left(t\right)+U_{C}\left(t\right)$$

is composed of the sensor signal $U_{S}\left(t\right)$ representing the mechanical movement of the beam:

(4) $$U_{S}\left(t\right)={\hat{U}}_{S}\times cos\left(2\pi ft+\varphi_{S}\right)+{\bar{U}}_{S}$$

and the parasitic signal $U_{C}\left(t\right)$ caused by a direct galvanic, capacitive, thermal, etc. coupling of the electrical actuation signal to the Wheatstone bridge. To subtract the $U_{S}\left(t\right)$ from $U_{S,M}\left(t\right)$, an EAM (Electromechanical Amplitude Modulation) has been created, in which the $U_{S,M}\left(t\right)$ is multiplied with a reference signal $U_{ref,EAM}$ related to the supply voltage of the Wheatstone bridge of the PCM:

(5) $$U_{0,EAM}={\hat{U}}_{0}\times cos\left(2\pi f_{WB}t\right)$$

with an additional gain. Here, ${\hat{U}}_{0}$ and $f_{WB}$ denote the amplitude and frequency of the EAM. The phases $\varphi_{S}$, ∆θ and the amplitudes ${\hat{U}}_{S}$, ${\hat{U}}_{A}$ of sensor and actuation signal, respectively, are measured by the I/Q demodulator. The parameters $\varphi_{S}$ and ${\hat{U}}_{S}$ can be kept constant at set points by adjusting ∆θ and ${\hat{U}}_{A}$ with the help of a PLL (Phase-Locked-Loop) and an AGC (Automatic Gain Control), respectively. In this way, the contact resonance frequency can be tracked. The DC component ${\bar{U}}_{S}$ of the sensor signal, representing the contact force, is kept constant by adjusting the *z*-position of the PCM by an FCL (Force Control Loop), achieving a topography tracking of the sample. For further details, see [34].

### 2.1. Electrothermal/Piezoelectric Actuation

**Figure 2:**
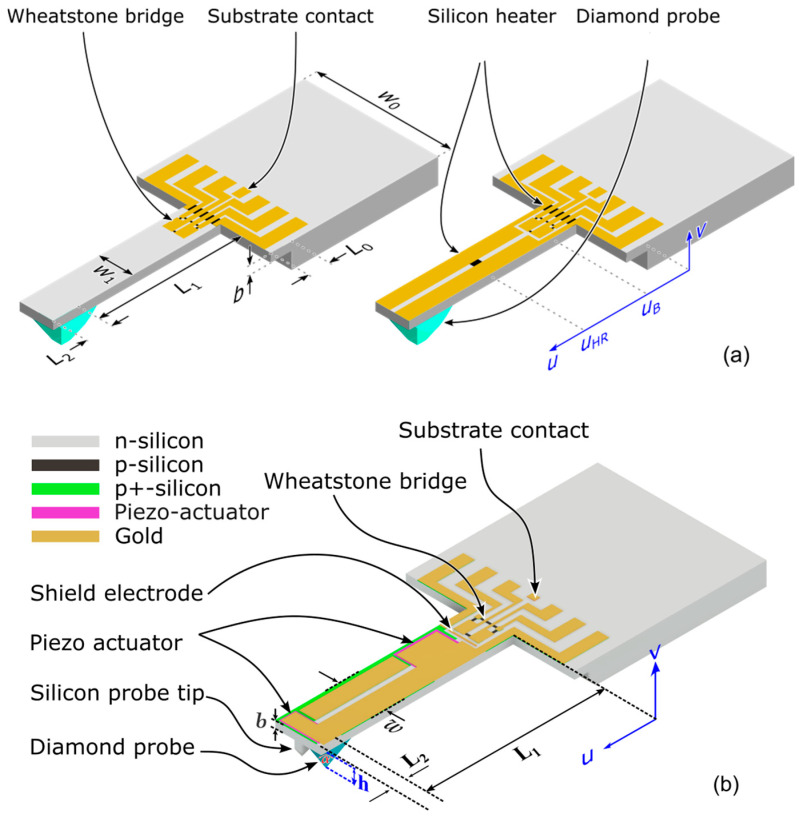
Schematic of electrothermal-actuated (**a**) and piezoelectric-actuated (**b**) piezoresistive microprobe (ETA-PCM and PEA-PCM).

The PCM is made from *n*-type silicon (100) wafers. Schematics of the sensor with integrated electrothermal (ETA) or piezoelectric actuators (PEAs) are shown in Figure 2, respectively. In the former, resistors are located at one or two positions on the cantilever, which are excited by a superposition of AC and DC components, as shown in Equation (1). For the current commercial PCM, CAN50-2-5, a unipolar voltage has to be supplied to the Wheatstone Bridge, so a substrate contact has been added in the new design to avoid current leakage caused by an AC voltage supply, which, with the EAM introduced above, the $U_{S}\left(t\right)$ can be abstracted from $U_{S,M}\left(t\right)$ without $U_{C}\left(t\right)$ (see Section 2.4).

For the ETA-PCM, the corresponding Joule power is:

(6) $$P=\frac{U_{A,thermal}^{2}}{R}=\frac{1}{2R}\left[2{\bar{U}}_{A}^{2}+{\hat{U}}_{A}^{2}+4{\bar{U}}_{A}{\hat{U}}_{A}cos\left(2\pi ft\right)+{\hat{U}}_{A}^{2}cos\left(4\pi ft\right)\right]$$

which generates local heating, resulting in a strain/stress gradient across the cantilever height and thus to a bimorph extension/compression of the upper regions of the cantilever along its axis, leading to vertical deflection. The DC component of *P* given by $2{\bar{U}}_{A}^{2}+{\hat{U}}_{A}^{2}$, includes the AC voltage amplitude. Therefore, an additional controller is required to keep the average power constant. As a result, the ETA actuators lead to sustained warming and, thus, deflection of the beam. Furthermore, using two separate resistors instead of one single resistor in regions of maximum strain/stress on the cantilever will improve the preferred excitation of a specific vibration mode.

As an alternative to ETA, a layer of piezoelectric material deposited on the cantilever is considered for vibration excitation in PEA-PCM. It is contacted at the underside with highly doped silicon and at the top with evaporated metal. Depending on the orientation of the applied field, the actuators expand or contract throughout the length of the beam due to the inverse piezoelectric effect, which results (as for the ETA) in a strain/stress gradient across the cantilever height and, thus to a bimorph deformation of along its axis leading to vertical bending of the beam [43]. The polarity of strain/stress is determined by the sign of the applied voltage/electric field. Here, the electrodes of adjacent actuators can be designed so that the polarity of the applied electric field is opposite, see Figure A6 in Appendix F. Thereby, specific vibration modes can be effectively stimulated. Furthermore, different from ETA (see Equation (2)), piezoelectric actuation (PEA) only requires AC excitation, i.e., no DC component is needed.

The beam’s movement, i.e., deflection and vibration, is measured using a piezoresistive Wheatstone bridge, which is located close to the clamped end of the beam where strain/stress is largest. It consists of four resistive elements, whose respective resistances change when stress is applied. This leads to a change in output voltage, which is proportional to the change in stress. To reduce interference between the output signal of the Wheatstone and the actuation signal of the actuators, a shield electrode is positioned between both. A silicon probe tip may be integrated into the sensor on the bottom surface of the cantilever. If required, an additional diamond tip can be glued behind the silicon tip. Both types of PCM are modeled in this work, and critical manufacturing steps are prepared.

To analytically analyze the mechanical behavior of the cantilever, it is divided into two regions: the region from clamping to the probe tip with length $L_{1}$ and the region from tip to the free end with length $L_{2}$. The entire cantilever has a uniform width *w* and a uniform thickness *b*. A probe tip with a height *h* is positioned at the cantilever bottom side near the free end. Parameter values are shown in Table 1.

### 2.2. Piezoresistive Wheatstone Bridge and Electronics

**Figure A2:**
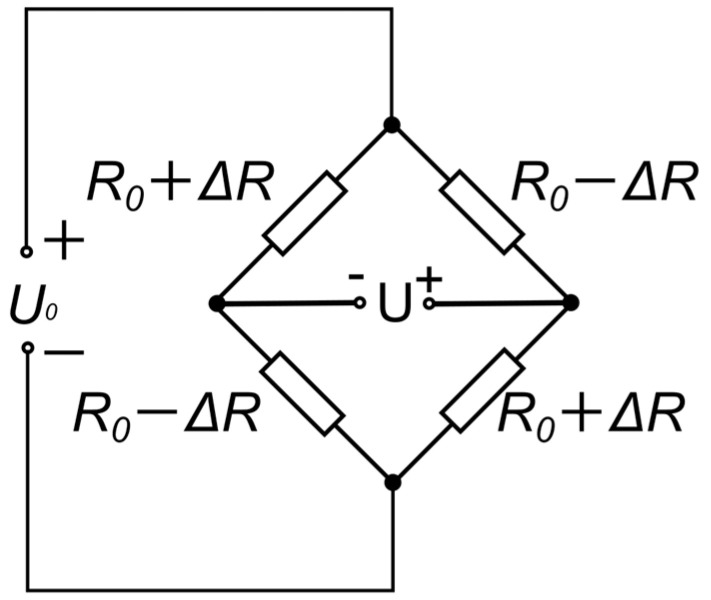
Circuit diagram of a Wheatstone bridge.

**Figure A3:**
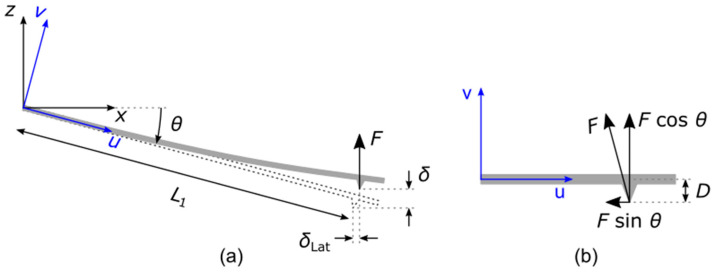
A sensor at angle of attack *θ* between the cantilever and a sample surface is deflected at its probe tip with the force *F*. (**a**) This leads to a deflection of *δ* in the direction of the force and to a deflection of *δ*_Lat_ perpendicular to the force. The resting position of the beam is shown by a dashed contour, the deflected shape by a filled area [55]. (**b**) The applied force generates a transverse force $Q_{m}\left(L_{0}+L_{1}\right)=Fcos\theta$ and a bending moment $M\left(L_{0}+L_{1}\right)=FDsin\theta$ in the beam at the position of the probe tip.

To construct the Wheatstone bridge, piezoresistive elements are integrated close to the clamped end of the beam, as shown in Figure 2; the circuit used here is depicted in Figure A2. In this circuit, all resistive elements have the same nominal resistance due to their identical size. When a force is applied to the probe tip, the beam deflects vertically, and normal stress is induced in the resistive elements, which leads to a change in resistance due to the piezoresistive effect: two resistive elements are compressed longitudinally, two are compressed transversely (for details see Appendix A and Appendix B). They are subjected to a nearly uniform normal stress $\sigma_{top,0}$ under vertical cantilever deflection (see Appendix B), as shown in Figure A3. Therefore, according to Equations (A14) and (A15), the resulting resistance changes of *p*-type resistors on a <110>-oriented cantilever can be approximately taken as identical for all resistive elements, and the output voltage U of the Wheatstone bridge is approximately given by:

(7) $$U=U_{0}\frac{∆R}{R_{0}}=U_{0}\frac{\pi_{44}}{2}\sigma_{top,0},$$

with the supply voltage $U_{0}$ and the piezoresistive matrix element $\pi_{44}$. Obviously, U does not depend on the specific resistance of the piezoresistive elements and is therefore prone to temperature-related fluctuations only via $\pi_{44}$.

In reality, however, the Wheatstone bridge is spatially extended. Thus, the local distribution of mechanical stress, both along the cantilever axis (in the *u* direction, see Figure A3) and its thickness (in the *v* direction, see Figure A3) have an effect on U. In addition, the carrier concentration across the resistance elements has a depth profile characteristic of the selected doping process, causing a spatial variation of resistivity and $\pi_{44}$ [43]. In the present study’s PCM, however, the thickness and length of the resistance elements are significantly smaller than the cantilever thickness and length, respectively, and for analytical modeling, we can assume the volume of the bridge is constricted to a point.

Sensor sensitivity is determined by the piezoresistive coefficient $\pi_{44}$, whose value is related to doping concentration. So, it is important to search for a suitable doping method to fabricate the Wheatstone bridge, which will be reported in detail below. The resistors of the Wheatstone bridge are covered by a metal layer for protection against distortion by ambient light and for reducing a temperature gradient across the bridge caused by the heating resistor in case of ETA, which may lead to a parasitic-feedthrough effect (see Section 2.4).

### 2.3. Beam Vibration

A vibration model is described in the following for design optimization of the PCMs and can also be used to evaluate measured data of their static and dynamic behavior. For this purpose, the modeling of freely oscillating cantilevers is addressed first, which is then extended to include the contact of the probe tip with a sample surface. The shape of the resulting cantilever deflection is calculated using the integration method [36] from the boundary conditions of the acting forces and moments at both ends of the cantilever, as well as conditions at the transition between the length segments 1 and 2 (see Table 1 and Figure 2). Since the applied force may be tilted with respect to the *z*-axis, i.e., in a direction not perpendicular to the cantilever axis (*x*-axis), the *uv*-coordinate system is used, which is rotated with respect to the *xz*-coordinate system by an angle of attack *θ* according to Figure A3. For the details of the mathematical derivation, see Appendix B, Appendix C, Appendix D and Appendix E. According to such vibration models, the ETAs or PCMs can be placed at the corresponding area of cantilever to drive the cantilever to oscillate (A more detailed introduction is shown in Section 3.1 and Section 3.4.1).

#### 2.3.1. Flexural Resonance Vibrations

Under standard atmospheric pressure, internal damping [44] of vibrating silicon cantilevers is negligible compared to external damping *η* by the surrounding air [45,46]. Therefore, cantilever vibration is described by the equation of motion [47,48]:

(8) $$E_{m}I\frac{\partial^{4}v}{\partial u^{4}}\left(u,t\right)+\eta \rho_{m}wb\frac{\partial v}{\partial t}\left(u,t\right)+\rho_{m}wb\frac{\partial^{2}v}{\partial t^{2}}\left(u,t\right)=0,$$

where $E_{m}$ and $\rho_{m}$ are Young’s modulus and density, respectively, of the cantilever material (silicon), and *I* is the moment of inertia. The deflection of the beam is analyzed separately in the two segments of the cantilever (Table 1 and Figure 2):

(9) $$v\left(u,t\right)=R\left\{\underline{v}\left(u\right)e^{i\omega t}\right\},\underline{v}\left(u\right)=\left\{\begin{array}{l} \underline{v_{1}}\left(u\right):0\leq u\leq u_{1},\\ \underline{v_{2}}\left(u\right):u_{1}<u\leq u_{2}, \end{array}\right.$$

where *i* is the imaginary unit, *ω* = 2π*f* is the angular frequency, and $\underline{v_{1}}$ and $\underline{v_{2}}$ are the complex parameters of the beam vibration. With the wave number *q* determined via

(10) $$q^{4}=\frac{\rho_{m}wb}{E_{m}I}\left(\omega^{2}\mp i\eta \omega \right),$$

we obtain

(11) $$\underline{v_{1}}\left(u\right)=c_{1}cos\left(qu\right)+c_{2}cosh\left(qu\right)+c_{3}sin\left(qu\right)+c_{4}sinh\left(qu\right),$$

(12) $$\underline{v_{2}}\left(u\right)=c_{5}cos\left(qu\right)+c_{6}cosh\left(qu\right)+c_{7}sin\left(qu\right)+c_{8}sinh\left(qu\right).$$

The unknown coefficients $c_{1}$ to $c_{8}$ are uniquely defined by the boundary conditions at both ends of the cantilever. In this work, the transfer matrix method [49,50,51] is used to calculate the motion of both segments of the cantilever one after the other. Here, equations for the complex parameters $\underline{v_{1}}$ and $\underline{v_{2}}$ are set up without calculating the coefficients $c_{1}$ to $c_{8}$. The resulting vibration model now enables calculating the amplitude and phase of deflection, slope, bending moment (and thus also of mechanical stress/strain), as well as shear force at arbitrary frequencies *ω*. The used material parameters of the beam are listed in Table 2. The boundary conditions for bending moment and transverse force are derived in Appendix C.

#### 2.3.2. Contact Resonance Vibrations

**Figure 3:**
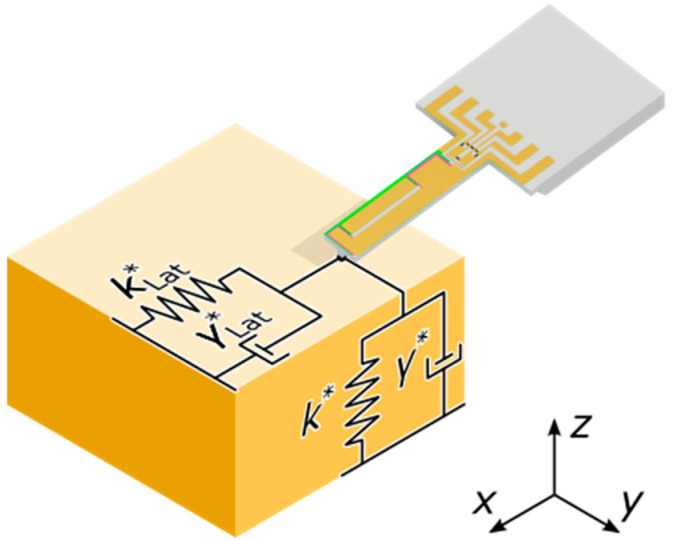
Interaction between the probe tip of a PCM and the sample surface of a viscoelastic material. Both lateral and vertical forces (relative to the surface orientation of the sample) are taken into account. Elastic behavior is modeled by vertical and lateral contact stiffness $k^{*}$ and $k_{Lat}^{*}$, viscous behavior is modeled by vertical and lateral contact damping $\gamma^{*}$ and $\gamma_{Lat}^{*}$.

When a PCM oscillates with its tip in contact with the surface of a sample, additional forces act on the beam related to vertical and lateral contact stiffnesses $k^{*}$ and $k_{Lat}^{*}$, as shown in Figure 3. Assuming that the vibration amplitude at the probe tip is significantly smaller than the static deformation of the sample and probe tip, $k^{*}$ and $k_{Lat}^{*}$ are almost constant. This results in a linear system of equations, which is evaluated using the transfer matrix method [49,55,56]. At too large oscillation amplitudes, nonlinear behavior is observed [57] which can be taken into account [58,59,60]. Since time-dependent behavior, in addition to static deflection, shall be analyzed, the viscosity of the contact materials is also considered (see Appendix D).

According to Hertzian contact mechanics [61,62], contact radius $a_{c}$ and contact stiffness $k^{*}$ and $k_{Lat}^{*}$ are given by [63]:

(13) $$a_{c}=\sqrt[{3}]{\frac{3Fr_{tip}}{4E_{m}^{{*}^{\prime }}}},$$

(14) $$k^{*}=2a_{c}E_{m}^{{*}^{\prime }},$$

(15) $$k_{Lat}^{*}=8a_{c}G_{m}^{{*}^{\prime }},$$

where $r_{tip}$ is the cone radius of the probe tip, $\underline{E_{m}^{{*}^{\prime }}}$ is the reduced storage modulus, and $\underline{G_{m}^{{*}^{\prime }}}$ is the reduced storage shear modulus.

To model viscous behavior, the interaction between the probe tip and the sample surface is extended by the contact-damping $\gamma^{*}$ and $\gamma_{Lat}^{*}$, as shown in Figure 3 [60,64]. The values of both elements are given by [63].

(16) $$\gamma^{*}=\frac{2a_{c}E_{m}^{{*}^{\prime \prime }}}{\omega },$$

(17) $$\gamma_{Lat}^{*}=\frac{2a_{c}G_{m}^{{*}^{\prime \prime }}}{\omega },$$

where $E_{m}^{{*}^{\prime \prime }}$ is the reduced loss modulus and $G_{m}^{{*}^{\prime \prime }}$ is the reduced loss shear modulus. The forces acting on the probe tip are considered by changed boundary conditions at the free end of the cantilever change compared to the free oscillating case, while they remain the same at its clamping (Appendix E) [65].

### 2.4. Fano Resonances

**Figure 4:**
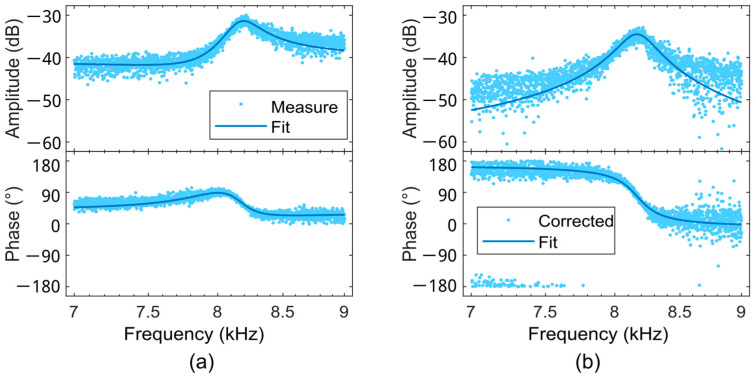
Measured spectral line shapes of amplitude (**upper**) and phase (**lower**) around resonance before (**a**) and after removing a Fano-resonance distortion (**b**).

Ideally, the measured frequency response from the piezoresistive Wheatstone bridge should represent the mechanical movement of the beam with a Lorentzian peak line shape with phase fall over 180°, but as shown in Figure 4a, measured resonance curves often show Fano-resonance behavior. This can be explained by the fact that a parasitic signal is superimposed on the measured sensor signal, which is due to galvanic [66,67], capacitive [68,69], and thermal [70] coupling of the electrical actuation into the measured signal. To describe the measured behavior, an electrical equivalent circuit [68,71] or an empirical model function [51] can be used and fitted to the measured data. In this work, an empirical model function is used. With the excitation signal $U_{A}(t)$, the measurement signal $U_{S,M}(t)$, the sensor signal $U_{S}(t)$, and the parasitic signal $U_{C}(t)$:

(18) $$U_{A}\left(t\right)=R\left\{\underline{U_{A}\left(\omega \right)}e^{i\omega t}\right\}$$

(19) $$U_{S,M}\left(t\right)=R\left\{\underline{U_{S,M}\left(\omega \right)}e^{i\omega t}\right\}$$

(20) $$U_{S}\left(t\right)=R\left\{\underline{U_{S}\left(\omega \right)}e^{i\omega t}\right\}$$

(21) $$U_{C}\left(t\right)=R\left\{\underline{U_{C}\left(\omega \right)}e^{i\omega t}\right\}$$

the sensor’s transfer behavior is described by [67,68]:

(22) $$\frac{\underline{U_{S,M}\left(\omega \right)}}{\underline{U_{A}\left(\omega \right)}}=\frac{\underline{U_{S}\left(\omega \right)}+\underline{U_{C}\left(\omega \right)}}{\underline{U_{A}\left(\omega \right)}}=\frac{\omega_{res}^{2}G_{res}}{{\left(i\omega \right)}^{2}Q_{res}+i\omega \omega_{res}+\omega_{res}^{2}G_{res}}\times e^{i\varphi_{delay}}+G_{C}\times e^{i\varphi_{C}},$$

where $\omega_{res}$ is the resonance angular frequency, $G_{res}$ is the ratio of sensor amplitude to excitation amplitude in resonance, $Q_{res}$ is the quality factor, $G_{C}$ is the ratio of parasitic signal amplitude to excitation amplitude and $\varphi_{C}$ is the phase shift between the parasitic signal and excitation signal, $\varphi_{delay}$ is the phase delay caused by signal propagation and response times of the measuring system components.

Figure 4a shows a measured PCM vibration spectrum around resonance, revealing asymmetric line shapes of amplitude and phase, which is modeled by fitting using Equation (22). After subtracting the parasitic component $\underline{U_{C}}\left(\omega \right)$, the line shapes of the amplitude and phase of a single harmonic oscillator are obtained (Figure 4b).

Parasitic feedthrough can be expected, especially for the ETA-PCM, even though the resistors of the Wheatstone bridge are covered by a metal layer to reduce a temperature gradient across the bridge caused by the heating resistor. The remaining (inductive, capacitive, thermal) feedthrough of the actuation signal into the Wheatstone bridge can be reduced using the model transfer function of Equation (22) given by the Fano-resonance effect [52,53]. PLL measurements with signals affected by parasitic feedthrough are enabled by electromechanical amplitude modulation (EAM) of the Wheatstone bridge supply voltage $U_{0}$. Here, instead of a pure DC value, $U_{0}$ is additionally AC-modulated at frequencies of around four times the sensing bandwidth and peak values of up to 10 V. For the PEA-PCM, without a DC component of the Wheatstone-bridge supply, the parasitic feedthrough will be reduced, so better results with the current homemade EAM can be expected.

## 3. Results and Discussion

### 3.1. Design Optimization of Electrothermal-Actuated Piezoresistive Microprobe

**Figure 5:**
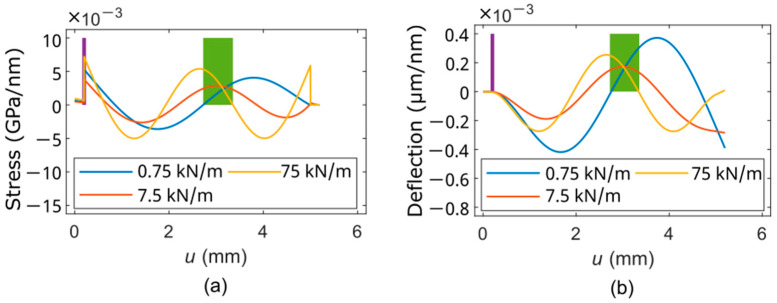
Eigenforms (**a**) of the third vertical bending mode and related strain distribution (**b**) along the surface of the cantilever (Table 1) are calculated using the described cantilever vibration model under varied contact stiffness. The angle of attack and the probing force are 0° and 10 µN, respectively. The optimal positions of the heaters are indicated by the purple and green bars, respectively. *u* is the position along the cantilever axis in the rotated coordinate system (see Figure A3).

For optimum design of a PCM with integrated actuators, their number and positions have to be adapted to a specific vibration mode. For this purpose, the eigenforms of the interesting vibration modes are calculated analytically under different contact conditions using the cantilever model described in Section 2.3. Optimum positions of the actuators are regions where the excited strain/stress in the top-surface region of the beam given by the eigenform shows a maximum for relevant ranges of contact stiffness (and damping). Furthermore, it is necessary to ensure that the actuators can be operated using an electrical signal of the same polarity. Eigenmodes higher than four are not considered, as this would reduce the actuator length too much, which in turn would affect the vibration amplitude. Using ETA, a maximum sensitivity can be expected with the third eigenmode at a contact stiffness of 7.5 kN/m. This corresponds at a probing force of 10 µN to a material with Young’s modulus of 60 MPa, which is in the range given by metals and ceramics. Excitation at *k*_c_ of 0.75 kN/m and 75 kN/m can be expected as well, but less effective. Under these conditions, two heaters are appropriate and best positioned, as shown in Figure 5.

### 3.2. Fabrication of Electrothermal-Actuated Piezoresistive Microprobe

**Figure 6:**
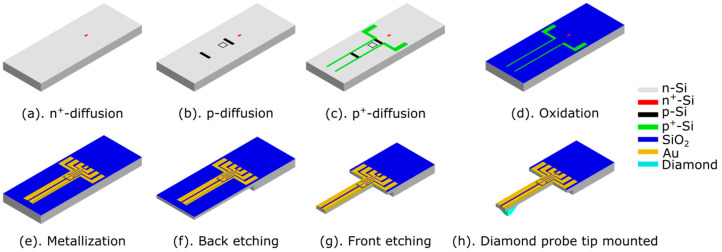
Manufacturing process of a PCM with integrated thermoelectric actuators.

Figure 6 shows a schematic of the fabrication process of the PCM with integrated thermoelectric actuators (ETAs). The process basically follows a standard in-house protocol [54]. It begins with an approximately 300 μm-thick wafer of *n*-doped (100) silicon (Siegert Wafer GmbH, Aachen, Germany), which is cleaned and then *n*^+^-doped in selected areas by phosphorus diffusion from a spin-on-deposited (SOD) silica source (a). This enables an Ohmic contact with the substrate, which helps to form a low-leakage *p*-*n* junction of the *p*-doped piezoresistive Wheatstone bridge to the *n*-type bulk. Here and in the following diffusion steps, the thermal oxide is used as a mask material, which is lithographically patterned before silica deposition and removed after diffusion. Thermal oxidation is performed at 1100 °C in a combined dry/wet process [54]. The *p*-doped elements of the piezoresistive Wheatstone bridge and the ETAs are then realized by boron diffusion from SOD silica (b). As illustrated in (c), those sites that are then contacted by highly *p*-doped (*p*^+^) regions formed in a second boron diffusion (from SOD silica). Once again, the surface is thermally oxidized to prevent short circuits caused by the following metallization. For Ohmic contact to the *n*^+^-and *p*^+^-doped silicon, holes are formed in the silicon dioxide (d) before the metallization by a layer stack of 30 nm chromium and 300 nm gold using electron-beam evaporation (e). Subsequently, the wafer is etched from its backside to the desired thickness of the cantilever beam by fluorine-based deep reactive ion etching at cryogenic temperature (cryoDRIE) (f) followed by cryoDRIE from the top surface (g). Finally, a diamond tip is glued manually onto the bottom surface of the cantilever close to its free end.

### 3.3. Contact Resonance Measurements of Electrothermal-Actuated Piezoresistive Microprobe

**Figure 7:**
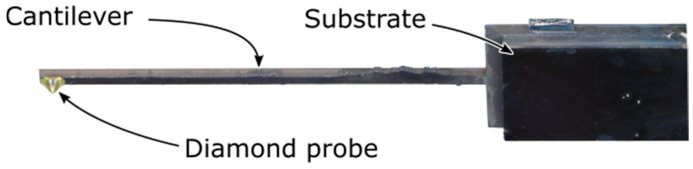
Bottom-side view of a fabricated PCM with an integrated ETA (top side, not visible here) and a glued diamond tip at the bottom side.

Figure 7 shows in a bottom-side view the photograph of a completed PCM comprising a glued diamond tip and an integrated ETA (at the top side, not visible here). Under excitation, we find the free fundamental bending mode of this PCM at 5.98 kHz ± 4 Hz (corresponding to a cantilever thickness of 110 µm) with a large quality factor of *Q* = 744 ± 136 Hz. The higher modes exhibit *Q* factors of almost an order of magnitude lower value. Using this PCM, CR measurements are then performed with bulk Al and a thin polymer layer on a glass substrate.

**Figure 8:**
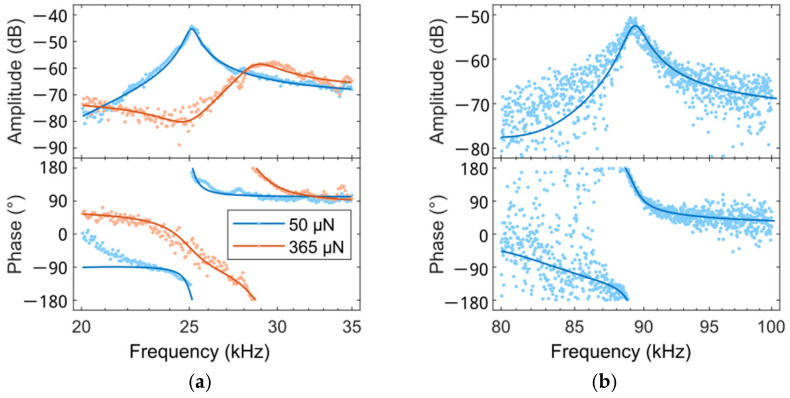
CR-measurements in the first (**a**) and second (**b**) modes using a PCM with integrated ETA and a diamond tip in contact with bulk Al (at a tilt angle of 0° and an actuation voltage of AC_peak_/DC = 2.5 V/2.5 V). The Wheatstone bridge is operated using an AC_peak_ supply of 10 V at 2 MHz (**a**) or 3 MHz (**b**). The contact forces are 50 µN/365 µN (**a**) and >1 mN (**b**). The light-colored circle points are measured via our homemade CRI setup. The superimposed lines are obtained by fitting using a Fano-resonance line shape (Equation (22)).

CR measurements using the PCM with integrated ETA operated using EAM with a milled surface of solid aluminum material are shown in Figure 8a, exhibiting fundamental-mode spectra, which are well fitted using Fano-resonance line shapes (Equation (22)). Resonance frequency shifts towards higher values, but amplitude decreases with increasing contact force, showing a strong asymmetry. The second bending mode can also be detected, as shown in Figure 8b. Here, however, noise is greatly increased to an effective value of ~0.3 mV at the selected modulation frequency of the Wheatstone bridge supply voltage of 3 MHz, which is reduced to around ~0.1 mV by lowering the modulation frequency to 2 MHz. The resonance curves are well described by the Fano-resonance line-shape fit (Equation (22)), i.e., CR measurements show a signal-to-noise ratio in both the first and second modes sufficient for the operation of the PCM using PLL combined with real-time correction.

**Figure 9:**
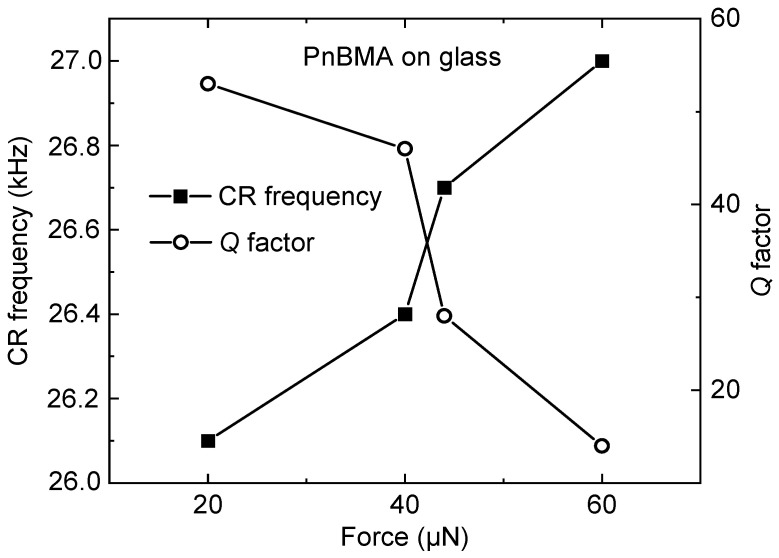
CR frequency and *Q* factor of the first mode using a PCM with integrated ETA and diamond tip in contact with a PnBMA layer on glass [72,73].

Figure 9 displays measurements of viscoelastic samples, including a thin polymer film. For a poly (n-butyl methacrylate) (PnBMA) layer on glass, the contact-resonance frequency increases with increasing probing force while the *Q* factor decreases [72,73].

**Figure 10:**
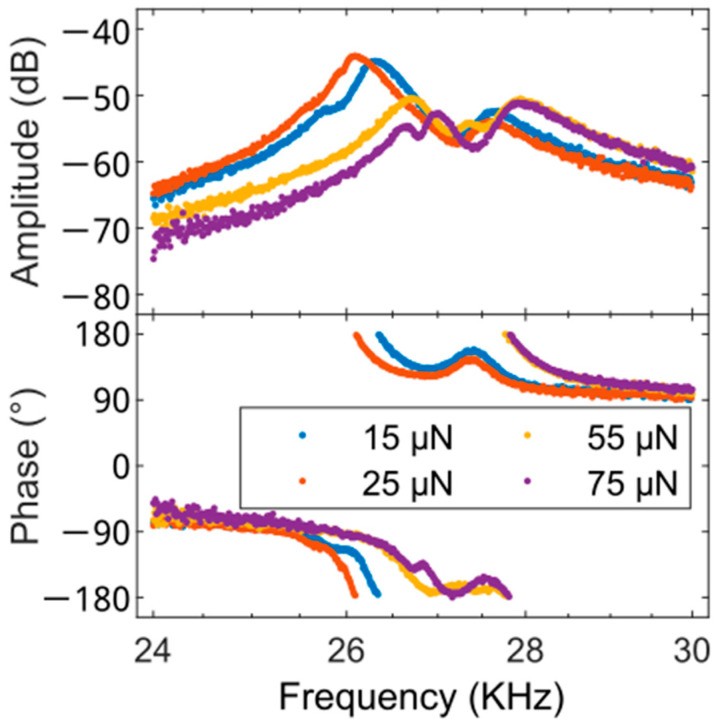
Amplitude and phase of the first CR mode with the PnBMA layer on glass using EAM of the Wheatstone bridge with an AC frequency of 2 MHz and a peak voltage of 10 V showing unidentified peaks around the fundamental CR mode.

Further, unidentified signatures are detected here, which were not observed in measurements of comparable samples using external piezoelectric actuators [48]. This effect is visible using the PCM with ETA, even if the Wheatstone bridge is operated using EAM. Figure 10 shows the amplitude and phase of the first CR mode with the PnBMA layer on glass using EAM with an AC frequency of 2 MHz and a peak voltage of 10 V.

EAM combined with Fano-resonance line-shape fitting is suitable for CR measurements using a PCM with integrated ETA. Nevertheless, it still suffers from severe drawbacks. First, continuous heating of the cantilever occurs, which causes considerable tip deflection (up to ~2.5 µm at 3.1 V effective heating voltage). Furthermore, the DC component of the excitation power of the ETA depends on the AC excitation amplitude, too (see Equation (1)). In consequence, automatic gain control (AGC) of the AC amplitude cannot be used since it leads to temporal variations in cantilever deflection and, thus, contact force. Therefore, in order to set the contact force precisely, the average heating voltage has to be controlled using an additional loop. Moreover, due to the non-linear dependence of the cantilever deflection on heating voltage, dynamic excitation is related to both the AC excitation amplitude and the DC component of the excitation voltage (see Equation (1)). This also applies to the parasitic feedthrough between the actuator and the Wheatstone bridge, which means it is not proportional to the electrical excitation amplitude. Thus, the Fano-resonance line-shape approach is not applicable unless the AGC is deactivated, i.e., the vibration amplitude is constant. Finally, measurements using an ETA-PCM on viscoelastic polymers reveal unidentified peaks around the CR frequency. Here, a more effective actuation of a specific eigenmode may be expected if two separate heating resistors instead of only one ETA are used.

### 3.4. Design Optimization of Piezoelectric-Actuated Piezoresistive Microprobe

To overcome the shortcomings of the ETA described above, the piezoelectric actuators (PEAs) are expected to deliver better performance since, ideally, temperature drift by Joule heating of the cantilever can be neglected, and no DC component is necessary for the actuation signal, enabling both AGC and a force-control loop using the AC/DC output of the piezoresistive Wheatstone bridge.

#### 3.4.1. Analytical Modeling of Cantilever Eigenmodes

**Figure 11:**
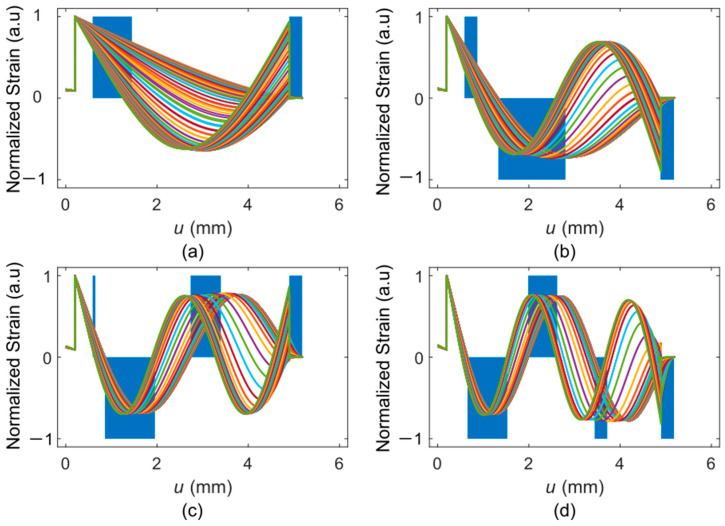
Strain along the surface of the cantilever in the eigenforms of the (**a**) first, (**b**) second, (**c**) third, and (**d**) fourth vertical bending mode, calculated for a diamond-tip sensor with contact stiffness between 0 and 100 kNm^−1^ and contact damping between 0 and 1 mNsm^−1^. The blue bars indicate the optimum positions and polarities of the actuators.

**Figure A6:**
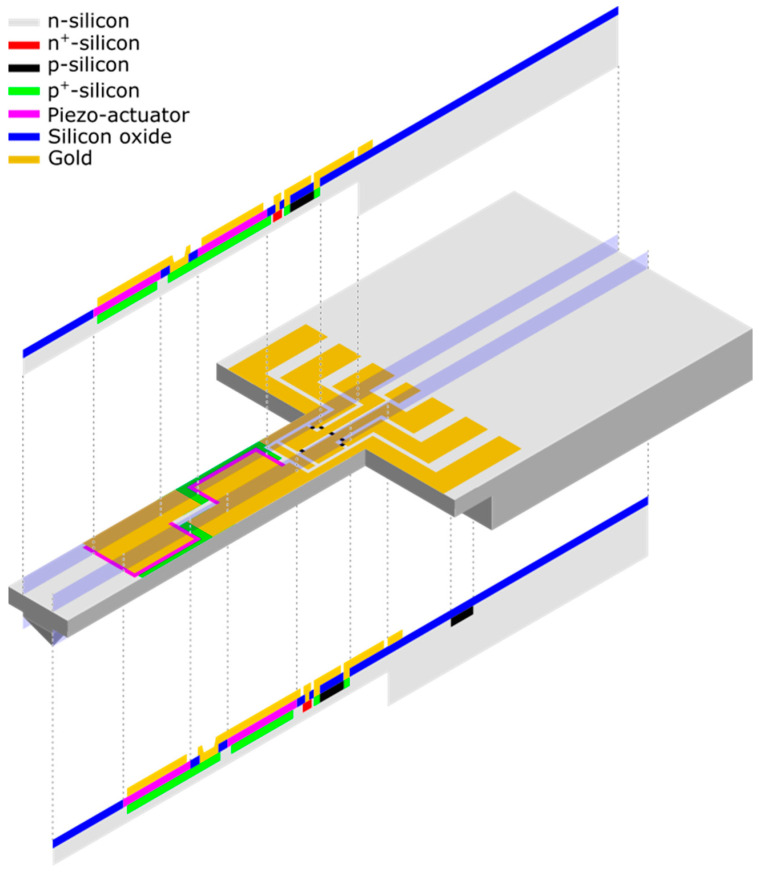

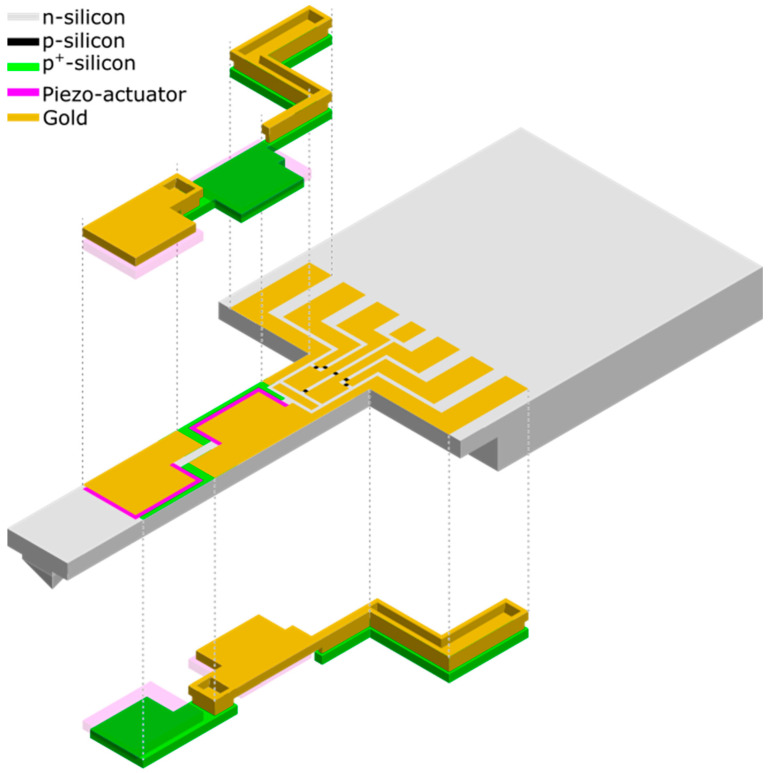
Layout of PCM with integrated PEAs.

Similar to the ETA-PCM introduced in Section 3.1, the number and positions of the actuators of the PEA-PCM are selected via the cantilever vibration model introduced in Section 2.3. Figure 11 shows the strain distributions along the surface of the cantilever excited in the first (a), second (b), third (c), and fourth (d) vertical-bending eigenmode and corresponding optimal positions of actuators modeled for a range contact stiffness and damping values. Two actuator positions in positive stress/strain segments are optimal for the first eigenmode, while further positions can be selected for the higher modes. Using PEA, the sign of the induced strain/stress can be set by the polarity of the applied voltage/electric field, which can be the same or opposite by a proper design of the actuators’ electrodes (see Figure A6). Thereby, all four bending modes can be effectively stimulated with properly positioned actuators (see Figure 11).

#### 3.4.2. Finite Element Analysis

**Figure 12:**
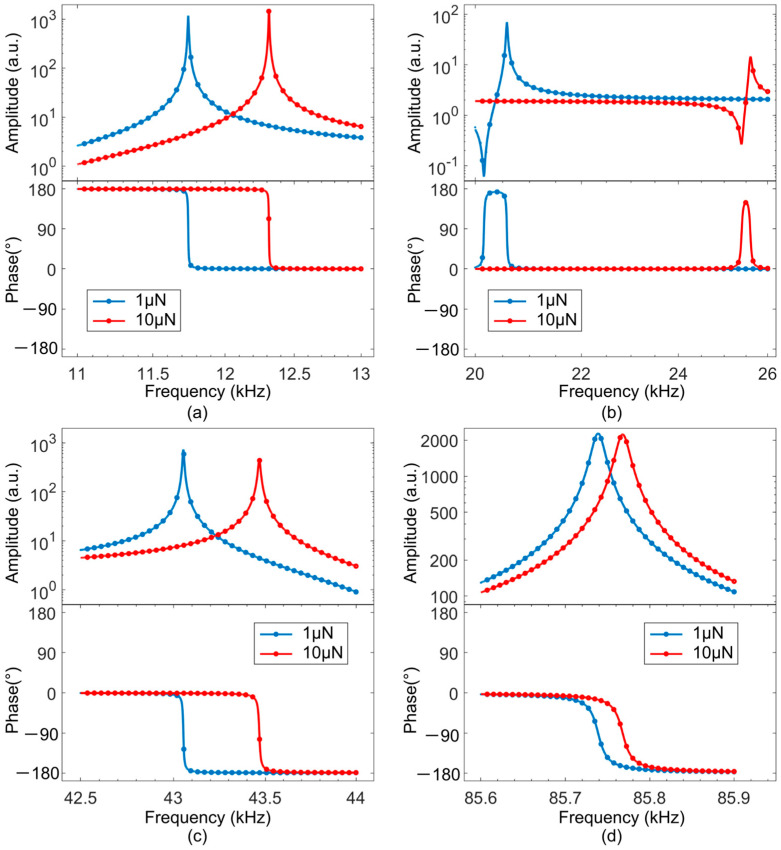
Finite element analysis (FEA) of the frequency response of a PCM with integrated piezo actuators positioned for preferential excitation of the first four vertical bending modes of the cantilever in contact with a sample of Young’s modulus of 10 GPa at angle of attack of 0°. The simulation results (light-colored circles) obtained by FEA of the first (**a**), second (**b**), third (**c**), and fourth (**d**) bending modes fitted using a Fano-resonance line shape are shown as colored lines (Equation (22)).

According to the above, the number and positions of the actuators are adapted to individual vibration modes by analytical modeling. To validate the concept, numerical simulations are carried out by finite element analysis (FEA) with a PCM with integrated PEAs, which are optimized for exciting the first four vertical bending modes; for details, see Appendix G. The FEA results with the PCM in contact at an angle of attack of 0° with a sample of Young’s modulus of *E*_m_ = 10 GPa are displayed as light-colored circles in Figure 12. As expected from contact mechanics, resonance frequency shifts with increasing contact force towards higher values, which is largest for the second mode. The first and the fourth vibration modes (Figure 12a,d) are excited most efficiently, as expected from the selected positions of the PEAs. As revealed by Figure 11a,d, they correspond to the left and right most positions recommended for the fourth mode, which have consistent polarity of stress/strain, too, but with opposite signs resulting in a phase shift of 180°. Due to an inconsistent polarity of stress/strain expected by the selected actuator position, much worse excitation can be expected for the second bending mode.

Superimposed on the FEA, least-squares fits of a Fano-resonance line shape (Equation (22)) are shown as colored lines in Figure 12, from which we obtain CR frequency and quality factor (*Q* factor) of the PCM, as shown in Table 3. In the first mode, resonance frequency and *Q* factor increase with the contact force, while *Q* decreases in the higher modes. The highest *Q* factor is found for the third mode, the lowest in the second mode, which is only poorly excited in the selected case of actuator positions.

### 3.5. Fabrication of Piezoelectric-Actuated Piezoresistive Microprobe

The electrothermal actuator of the ETA-PCM is realized via boron diffusion simultaneously with the formation of the Wheatstone bridge. In contrast, PEA-PCM only uses the *p*^+^ contact-diffusion step for the bottom contact of the piezoelectric layer. In addition, it necessitates a further oxidation step for insulating the top contact of the piezoelectric actuator from the bottom contact. During this additional oxidation step, the already realized resistor elements, as well as their contact region, are affected by the consumption of doped silicon from the resistors’ surface and the diffusion of carriers deeper into the substrate. This will ultimately influence the configured Wheatstone bridge, necessitating an examination of the final doping profile to ascertain suitable doping parameters for the synthesis of the PEA-PCM.

#### 3.5.1. Phosphorus and Boron Diffusion

The piezoresistive Wheatstone bridge of the PCM converts mechanical strain into electrical signals, whose resistors are built by *p*-diffusion and *p*^+^-diffusion (for contact formation). The doping parameters for it have to be adopted to the boundary conditions by the manufacturing process. Meanwhile, the wires and bottom electrodes of PEAs are simultaneously built by the *p*^+^-diffusion step, while the substrate contact is realized by *n*^+^-diffusion. In this study, we use diffusion from a spin-on-deposited (SOD dopant source, which does not require dangerous gases, including related safety precautions, and is more environmentally friendly. Similarly to ion implantation, it allows area-selective doping, e.g., of shallow resistors and piezoresistors using lithographic patterning. Meanwhile, it is also the simplest, most widely available technique adaptable to different platforms, as indicated by its long history. During diffusion, there is minimal lattice damage, although excess dopants tend to aggregate at the surface. Nevertheless, unless a drive-in phase is incorporated into the manufacturing process, diffusion typically (but not always) results in lower cross-wafer uniformity than ion implantation, and the dopant concentration is fixed at the solid solubility concentration [43]. Correspondingly, sheet resistance and dopant depth profiles in resistive layers depend on diffusion temperature and time, which must be determined beforehand using monitor wafers.

**Figure 13:**
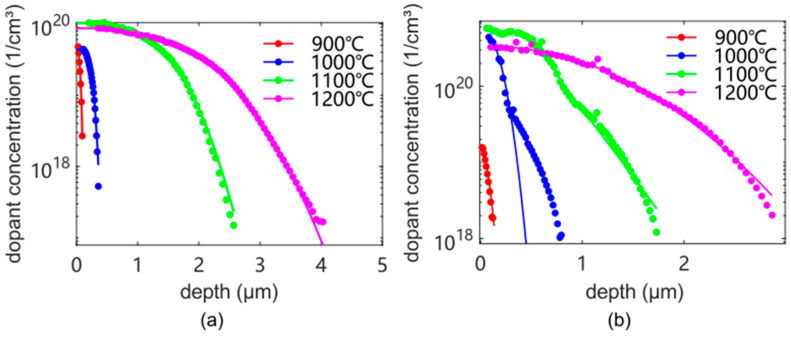
The electrically active dopant concentration profile of (**a**) boron dopant (PBF2.2DS, Filmtronics, Butler, PA, USA) and (**b**) phosphorus (P509, Filmtronics, Butler, PA, USA) in *n*-type silicon (100) (resistivity: 1–10 Ohm-cm, Siegert Wafer, Germany) at different processing temperatures for 30 min, in dependence on depth from the top surface. The solid dots are the experiment data measured using electrochemical capacitance-voltage (ECV) profiling. The solid lines are fits based on a series of Gaussian normal distributions to the experiment data (Equation (23)).

To confine the electric current to a resistor, it is electrically isolated from the bulk of the wafer using a low-leakage *p*-*n* junction. In an *n*-type silicon (100) wafer (resistivity: 1–10 Ohm-cm, Siegert Wafer, Germany), a boron *p*-type dopant (PBF2.2DS, Filmtronics, Butler, PA, USA) is introduced into silicon for resistors with defined dimensions and defined concentration with a minimum amount of surface damage. With opposite charges, the *p*-type and *n*-type dopants compensate one another. Hence, the junction depth is defined as the depth at which the impurities have the same concentration as the initial background concentration. A phosphorus dopant (P509, Filmtronics, Butler, PA, USA) is introduced at a specific site to improve Ohmic contact with the substrate. For both dopants, the concentration distribution is a function of processing conditions, especially temperature. To determine appropriate values, diffusion is performed (at atmospheric pressure with 4 L/min N_2_ (dry) and 0.5 L/min O_2_ (dry)) in the range of 900 °C, 1000 °C, 1100 °C, and 1200 °C, respectively, for 30 min. The details about the diffusion process are shown in Table A3 in Appendix H. The electrically active dopant concentration is measured via an electrochemical capacitance-voltage profiler (WEP, Furtwangen, Germany), as shown in Figure 13, in which the experiment data are the solid dots and solid lines fitted by a series of Gaussian normal distributions:

(23) $$y=\sum_{i=1}^{n}a_{i}e^{\left[-{\left(\frac{x-b_{i}}{c_{i}}\right)}^{2}\right]}$$

Here, *a* is the amplitude, *b* is the centroid (location), *c* is related to the peak width, and *n* is the number of Gaussian peaks to fit (1 ≤ *n* ≤ 8) [74]. In Figure 13, *y* and *x* correspond to carrier concentration and depths, respectively. The values of the above fitting parameters are shown in Table A4 in Appendix H. As expected, the dopant depth increases significantly with increasing temperature, and surface dopant concentrations at 1100 °C and 1200 °C are substantially higher than at 900 °C and 1000 °C.

To calculate junction depth and resistance, we use the PV Lighthouse calculator [74]:

(24) $$\rho_{sq}=\frac{1}{q\cdot \int_{0}^{z_{j}}\mu_{maj}\left(z\right)\cdot N\left(z\right)\cdot dz},$$

where $\rho_{sq}$ is the sheet resistance (Ω/sq) determined from the net ionized doping concentration $N\left(z\right)$ and the mobility $\mu_{maj}$ of the majority carriers. $z_{j}$ is the junction depth and q is the elementary charge of an electron. The net ionized doping concentration is defined as $N\left(z\right)={|N}_{A}\left(z\right)-N_{D}\left(z\right)|$, where $N_{A}\left(z\right)$ and $N_{D}\left(z\right)$ are the ionized concentrations of acceptor and donor atoms, respectively. In this study, boron atoms are the acceptors (introduced by diffusion), and phosphorus atoms are the donors (introduced by diffusion and background doping); the measured electrically active dopant concentration shown in Figure 13 corresponds to $N\left(z\right)$.

**Figure A8:**
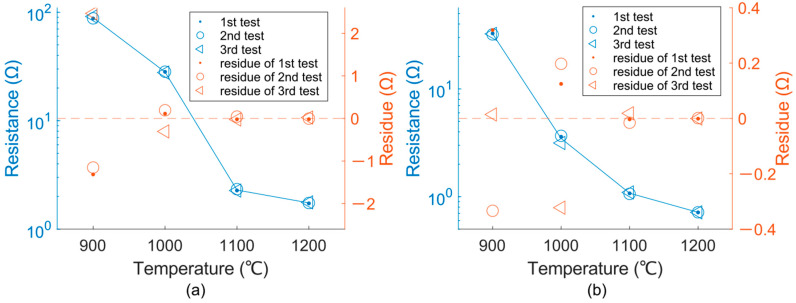
The resistance measured by four-point probing (FPP) of (**a**) boron dopant (PBF2.2DS, Filmtronics, Butler, PA, USA) and (**b**) phosphorus (P509, Filmtronics, Butler, PA, USA) diffusion profiles in *n*-type silicon (100) at different processing temperatures for 30 min. The blue points, circles and triangles describe the resistance values measured at three different locations on the sample. The red symbols describe the residues of each measurement with respect to the average value at each temperature.

Furthermore, the aforementioned samples are characterized using four-point probing (FPP) [75]. Figure A8 in Appendix H displays the measured data, which may be converted to sheet resistance by:

(25) $$\rho_{sq}=4.53236\;R_{FPP}$$

where $R_{FPP}$ is the average resistance measured for each temperature.

Table A5 and Table A6 in Appendix H display the obtained results in dependence on temperature *T*, which is consistent with the supplier’s data: as temperature rises while sheet resistance falls. However, at the same temperature, the experimental data are larger than the supplier data [58], which could be attributed to different diffusion conditions (furnaces, e.g.).

**Figure A9:**
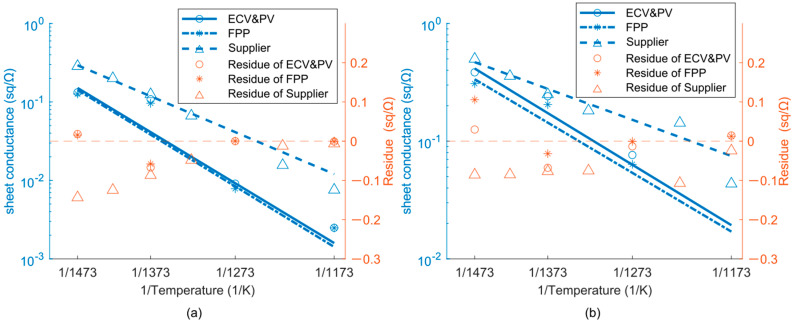
Sheet conductance of boron (**a**) (PBF2.2DS, Filmtronics, Butler, PA, USA) and (**b**) phosphorus (P509, Filmtronics, Butler, PA, USA) dopants in *n*-type silicon (100) at different doping temperatures. The blue triangles depict data from the supplier. The blue circles and asterisks depict data calculated from ECV [73] and FPP, respectively (Table A5 and Table A6). The lines are exponential fits of Equation (26), and the fitting parameters are shown in Table 4.

Dopant diffusion in the considered temperature range is a thermally activated process with an Arrhenius temperature dependence of diffusivity. The integral doping concentration is proportional to the square root of diffusivity [74]. Therefore, we analyze the diffusion experiments using

(26) $$\sigma_{sq}=k\cdot e^{-\frac{E_{a}}{2k_{B}T}},$$

where *σ_sq_* (=1/*ρ_sq_*) is the sheet conductance (assumed to be proportional to the integral doping concentration) in Figure A9 in Appendix H, *k* is a proportionality factor, *E_a_* is the activation energy of dopant diffusion, *k_B_* is the Boltzmann constant and T is the diffusion process temperature. The activation energies obtained by fitting Equation (26) to the data in Figure A9 in Appendix H are displayed in Table 4. We find good agreement between the values from ECV and FPP. Reasonable agreement is obtained for the activation energy of boron diffusion with respect to the analysis of the supplier’s data. In the case of phosphorus diffusion, ECV and FPP results show better agreement with published data than the supplier’s value. Further data on diffusion using PBF2.2DS are not available. We assign the observed deviations of *E_a_* to different conditions of solution spinning/curing and diffusion ambient (e.g., the N_2_/O_2_ ratio).

According to Appendix A, the sensitivity of the Wheatstone bridge is determined by the dopant concentration. To determine this, the expected piezoresistive coefficients at various processing temperatures are calculated based on Equations (A16) and (A17) in Appendix H. As shown in Table 5, at 1000 °C, the $\pi_{44}$ is maximum. We can thus expect that a Wheatstone bridge manufactured at 1000 °C will exhibit the greatest fractional change in resistance or voltage change and will be most sensitive to a mechanical input (e.g., by a force on the tip of the PCM). However, subsequent high-temperature steps (oxidations and *p*^+^ diffusion) in the fabrication process of the PCM will affect the doping profile and, thus, the final resistance and piezoresistive coefficient.

#### 3.5.2. Effect of High-Temperature Steps

The doping profiles obtained after diffusion are affected by subsequent high-temperature steps during PCM fabrication, i.e., the p+ diffusion at 1200 °C and additional oxidations (at 1100 °C) required to provide masks for area-selective doping of the resistor elements and piezoelectric ceramics deposition. Each of these high-temperature steps affects the doping profiles, sheet resistances, and junction depths generated before, e.g., during oxidation, silicon material from the wafer top is consumed, and the dopants are driven to larger junction depths. These effects must be taken into account while selecting the p and p+ diffusion parameters, e.g., to achieve a resistance of the Wheatstone Bridge resistors similar to 2.5 kΩ of the commercial CAN50-2-5.

**Figure 14:**
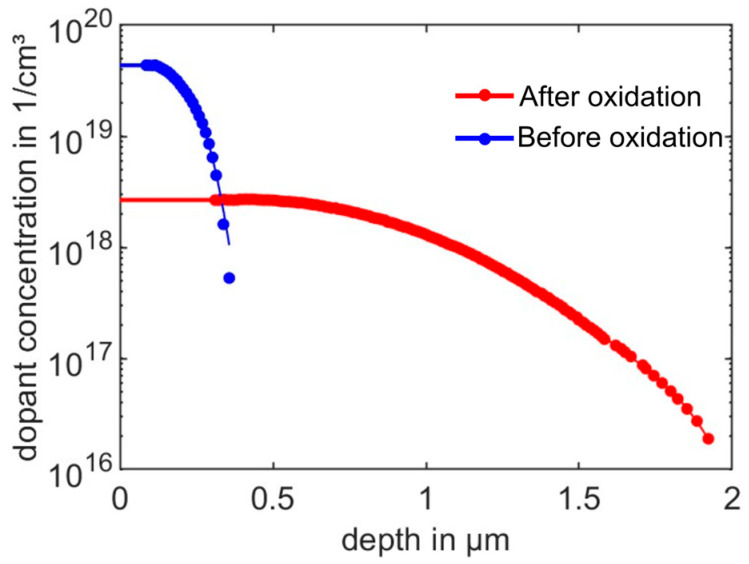
Dopant concentration profiles in silicon fabricated by boron diffusion at 1000 °C before and after two subsequent thermal oxidation steps for 105 min at 1100 °C. The filled circles are the experiment data from ECV. The solid lines are fits based on a series of Gaussian normal distributions to the experiment data by Equation (23). The fitting parameters are shown in Table A4 in Appendix H.

**Figure 15:**
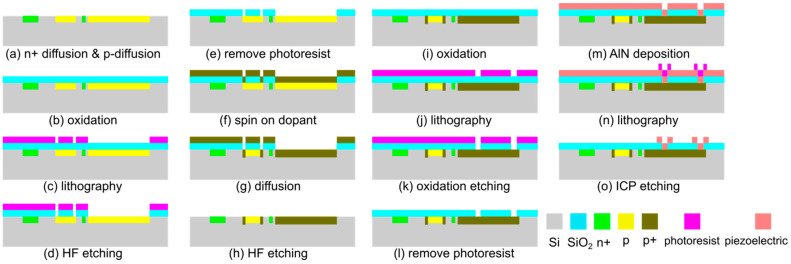
Fabrication process of a PCM with piezoelectric actuators.

Figure 14 shows the concentration profiles in silicon fabricated by boron diffusion for 30 min at 1000 °C before (corresponding to Figure 15a) and after two subsequent oxidations for 105 min at a temperature of 1100 °C (corresponding to Figure 15b,i). As expected, there is a decrease in the surface concentration but an increase in the junction depth. For the final doping profile, we calculate a sheet resistance, junction depth, surface doping concentration, and piezoresistive coefficient ($\pi_{44}$) of 193.78 Ω/sq, 1.91 µm, 1.62 × 10^18^ cm^−3^ and $1.08\;{\mathrm{GPa}}^{-1}$, respectively, which are considerably changed with respect to the values before oxidation (110.12 Ω/sq, 0.35 µm, 4.37 × 10^19^ cm^−3^, and $0.64\;{\mathrm{GPa}}^{-1}$). According to sheet resistance and dimensions, the expected resistance of the Wheatstone Bridge resistors increases from 0.88 kΩ before to 1.55 kΩ after oxidation. The Wheatstone Bridge resistance of the commercial CAN50-2-5 is 2.5 kΩ for reference. The larger piezoresistance coefficient of $\pi_{44}=1.08\;{\mathrm{GPa}}^{-1}$ will lead to higher sensitivity of the PCM. In contrast, the larger junction depth (representing the piezoresistor thickness) will cause a decrease in sensitivity due to the reduced mechanical stress away from the cantilever surface [43]. However, the measured junction depth of ~2 µm accounts for only ~4% of the designed cantilever’s thickness (50 µm). Under this condition, mechanical stress in the piezoresistors will nearly correspond to that at the cantilever surface. Therefore, the finite junction depth is expected to have only a minor effect on sensor sensitivity.

#### 3.5.3. Manufacturing Process

As already indicated in the preceding subchapters, the manufacturing process for PCMs with PEAs is shown in Figure 15. It is based on the protocol established for PCMs with ETA in Figure 6. The process starts with the cleaning of a 300 μm-thick wafer of *n*-doped (100) silicon, which is then *n*^+^-doped in selected areas by phosphorus diffusion from SOD silica at 1200 °C, then *p*-doping is performed by boron diffusion at 1000 °C in Figure 15a. For Ohmic contact to *p*-type silicon for the piezoresistive Wheatstone bridge and the bottom electrodes of the piezoelectric actuators, *p*^+^ doping is performed by a second boron diffusion at 1200 °C in Figure 15b–h. After further oxidation and patterning step (Figure 15i–l), AlN is deposed by magnetron sputtering and patterned by chlorine-based dry chemical etching (Figure 15m–o). Metallization, cantilever etching, and manual mounting of a diamond tip will be performed as described for the PCM with ETA in Figure 6e–h. While the steps Figure 15a–i already have been completed, AlN sputtering is still ongoing with assistance from Fraunhofer Institute for Electron Beam and Plasma Technology FEP, Dresden, Germany [78], which will be reported in a subsequent paper.

## 4. Conclusions

A piezoresistive cantilever microprobe (PCM) with an integrated electrothermal (ETA) or piezoelectric actuator (PEA) has been described, which is designed for viscoelastic-property surface imaging of workpieces using contact resonance imaging (CRI). Compared to commercial PCMs, which require external actuators, integrated actuators can directly excite specific eigenmodes of the cantilever. A substrate contact has been added in the new design for the AC voltage supply of the Wheatstone bridge to reduce parasitic signal influence via the EAM (Electromechanical Amplitude Modulation) in our homemade CRI system. For optimizing the number and positions of the actuators on the cantilever, specific contact-resonance (CR) vibration modes of the PCM are investigated via analytical modeling. Samples of PCMs with ETA are fabricated in a bulk-silicon micromachining process using diffusion from spin-on-deposited dopant sources, thermal oxidation for masking, electron-beam evaporation for metallization and fluorine-based deep reactive ion etching at cryogenic temperature for cantilever formation. Boron-doped resistors comprising high-concentration contact areas are realized simultaneously for the piezoresistive Wheatstone bridge and the ETA. In CR measurements of the completed ETA-PCM with bulk aluminum and thin-film-polymer samples, the expected force dependence of contact resonance frequency is observed. Parasitic feedthrough is reduced using electromechanical amplitude modulation (EAM) via a combined AC/DC supply of the Wheatstone bridge. EAM, in combination with a Fano-type line-shape analysis of CR, is shown to reveal material-specific signatures (single harmonic resonator) of contact stiffness and damping from the measured spectra. Nevertheless, unspecific peaks remain in the spectra of soft polymer samples, which we can relate to the non-linear dependence of the cantilever deflection on heating voltage since dynamic excitation is determined by both the AC and DC components of the excitation voltage. Therefore, PCMs with integrated piezoelectric actuators are considered as the next step. The design of a PEA-PCM and its performance in CR conditions are investigated using finite element analysis (FEA), providing the spectral characteristics of amplitude and phase output of the piezoresistive Wheatstone bridge when the cantilever tip is in contact with samples of specific viscoelastic properties. The FEA results were fitted using a Fano-resonance line shape to deliver resonance frequency and *Q* factor of the sample with a measured Young’s modulus of 10 GPa. Finally, the influence of an additional oxidation step on the doping profile of the Wheatstone-bridge resistors was analyzed, and suitable doping parameters for the PEA-PCM manufacture protocol were established.

## Figures and Tables

**Table 1:** Dimensions of the piezoresistive cantilever microprobe (PCM).

Parameter	Symbol	Value
Position of Wheatstone bridge	$u_{B}$	85 µm
Length of segment 1	$L_{1}$	4950 μm *
Length of segment 2	$L_{2}$	50 μm *
Width	*w*	200 μm [42]
Thickness	*b*	50 μm [42]
Height of silicon probe tip	*h*_tip,Si_	100 μm [38]
Opening angle of silicon probe tip	*α*_tip,Si_	40° [40]
Mass of silicon probe tip	*m*_tip,Si_	0.32 µg
Height of diamond probe tip	*h*_tip,Dia_	200 μm [32]
Opening angle of diamond probe tip	*α*_tip,Dia_	90° [32]
Radius of diamond probe tip	*r*_tip,Dia_	2 µm
Mass of diamond probe tip	*m*_tip,Dia_	18.9 µg

* Measured on CAN50-2-5 sensors.

**Table 2:** Mechanical properties of silicon with Young’s modulus given in the <110> crystal direction, the indentation modulus in the <100> direction, the shear modulus for shear stress in the {100} plane (with the shear strain perpendicular to it in the <100> direction), and Poisson’s ratio in the {100} plane.

Parameter	Symbol	Value
Young’s modulus	$E_{m}$	169 GPa [52]
Indentation module	$M_{m}$	165 GPa [53]
Shear module	$G_{m}$	79.6 GPa [52]
Loss factor	ζ	6 × 10^−5^ [54]
Poisson’s ratio	ν	0.28 [52]
Density	$\rho_{m}$	2330 kg·m^−3^ [54]

**Table 3:** Contact-resonance frequency and *Q* factor of the PCM with integrated piezo actuators in contact with a sample of Young’s modulus of 10 GPa determined by FEA and fitting using a Fano-resonance line shape (Figure 12).

Contact Force	1 µN		10 µN	
Vibration Mode	Resonance Frequency (kHz)	*Q* Factor	Resonance Frequency (kHz)	*Q* Factor
1	11,740	2290	12,310	2860
2	20,580	890	25,600	460
3	43,050	6090	43,470	3780
4	85,740	5490	85,770	5400

**Table 4:** The values of the exponential fitting parameters (Equation (26)) of sheet conductance vs. phosphorus and boron diffusion process temperatures.

Data Source	*p* Doping (Boron, PBF2.2DS)		*n* Doping (Phosphorus, P509)	
	*T* (°C)	*E_a_* (eV)	*T* (°C)	*E_a_* (eV)
ECV&PV	900–1200	4.52 ± 16.53	900–1200	3.04 ± 3.82
FPP	900–1200	4.57 ± 15.47	900–1200	2.96 ± 4.05
Filmtronics	900–1200	3.17 ± 0.51	900–1200	1.82 ± 0.68
[76]	-	-	840–990	3.2 ^(^*^)^
[77]	-	-	860–920	3.88 ^(^**^)^

^(^*^)^ Diffusion from P509 SOD silica source. ^(^**^)^ Diffusion from P508 SOD silica source.

**Table 5:** Surface dopant concentration and piezoresistive coefficient calculated using Equations (A16) and (A17) in Appendix H at *T* = 300 K for the different doping temperatures.

Temperature (°C)	Surface Doping Concentration (10^19^ cm^−3^)	$\mathrm{Piezoresistive}\;\mathrm{Coefficient}\;(\pi_{44}$/GPa^−1^)
900	4.78	0.6295
1000	4.37	0.6443
1100	10.4	0.4997
1200	8.66	0.5302

**Figure A1:**
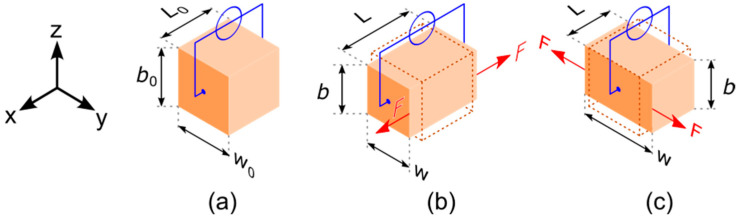
Deformation of an isotropic elastic body under applied normal force. (**a**) Body without external force, (**b**) Body with longitudinally applied normal force, (**c**) Body with transversely applied normal force.

**Figure A4:**
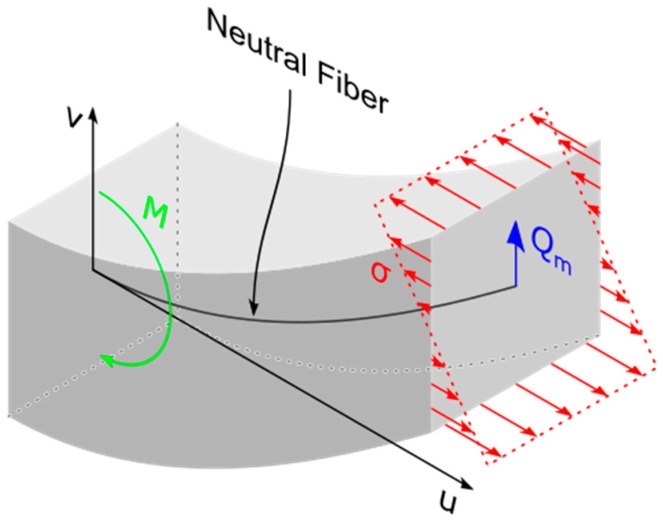
Shear force *Q_m_*, the bending moment *M* and the mechanical stress *σ* in the bend beam.

**Figure A5:**
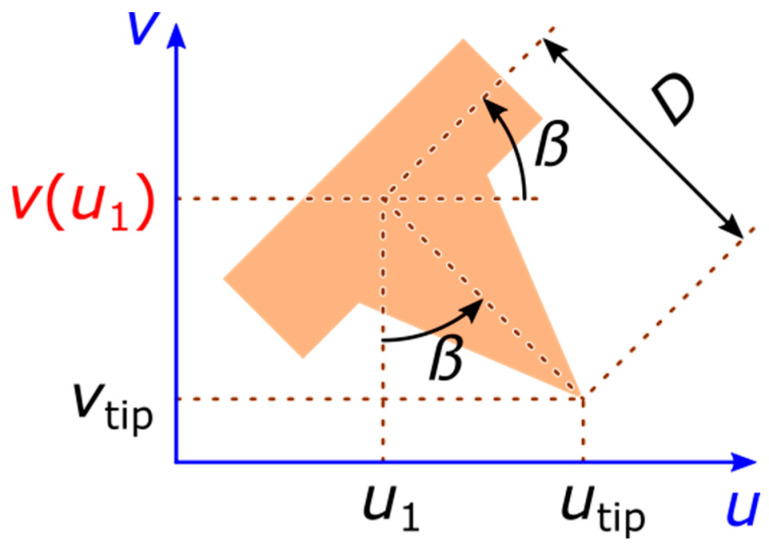
Position of the contact point of the deflected probe tip.

**Figure A7:**
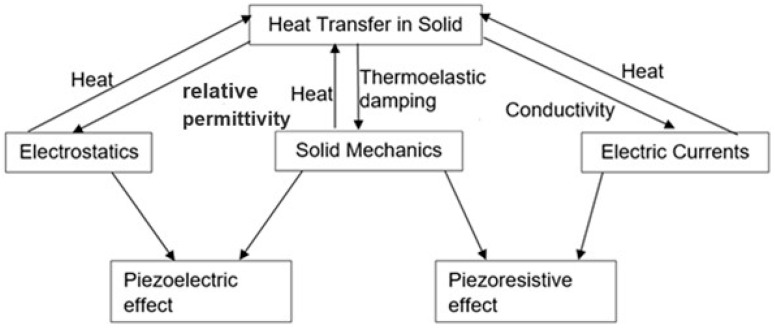
Bock diagram of used modules of COMSOL Multiphysics 5.4 for FEA.

## Data Availability

Data is contained within the article. The original contributions presented in this study are included in the article. Further inquiries can be directed to the corresponding authors.

## Abbreviations

**Parameter**	**Symbol**
Analog-to-digital converter	ADC
Automatic Gain Control	AGC
Contact resonance imaging	CRI
Digital-to-analog converter	DAC
Direct Digital Synthesizer	DDS
Diamond-like carbon	DLC
Electromechanical Amplitude Modulation	EAM
Electrothermal actuation	ETA
Force Control Loop	FCL
Finite element analysis	FEA
Personal Computer	PC
Piezresistive cantilever microprobe	PCM
Piezoelectric actuator	PEA
Phase-Locked-Loop	PLL
Spin-on-deposited	SOD
Serial Peripheral Interface	SPI
Variable Gain Amplifier	VGA
Reduced storage modulus	$\underline{E_{m}^{{*}^{\prime }}}$
Reduced storage shear modulus	$\underline{G_{m}^{{*}^{\prime }}}$
Actuation amplitude of actuation signal	${\hat{U}}_{A}$
DC component of actuation signal	${\bar{U}}_{A}$
Amplitude of sensor signal	${\hat{U}}_{S}$
DC component of sensor signal	${\bar{U}}_{S}$
Complex parameters of the beam vibration	$\underline{v_{1}}$, $\underline{v_{2}}$
Phase width	$B_{\theta (n)}$
Reduced loss modulus	$E_{m}^{{*}^{\prime \prime }}$
Young’s modulus	$E_{m}$
Ratio of sensor amplitude to excitation amplitude in resonance	$G_{res}$
Ratio of parasitic signal amplitude to excitation amplitude	$G_{C}$
Reduced loss shear modulus	$G_{m}^{{*}^{\prime \prime }}$
Shear module	$G_{m}$
Length of segment 1 of cantilever	$L_{1}$
Length of segment 2 of cantilever	$L_{2}$
Indentation module	$M_{m}$
Concentration of ionized acceptor atoms	$N_{A}\left(z\right)$
Concentrations of ionized donor atoms	$N_{D}\left(z\right)$
Quality factor	$Q_{res}$
Resistance measured by four points probes (FPP)	$R_{FPP}$
Supply voltage of the Wheatstone bridge with EAM	$U_{0,EAM}$
Supply voltage of the Wheatstone bridge	$U_{0}$
Actuation signal of PEA	$U_{A,piezo}\left(t\right)$
Actuation signal of ETA	$U_{A,thermal}\left(t\right)$
Parasitic signal	$U_{C}\left(t\right)$
Reference signal of EAM	$U_{ref,EAM}$
Measurement signal	$U_{S,M}\left(t\right)$
Sensor signal	$U_{S}\left(t\right)$
Actuation signal	$U_{A}(t)$
Contact radius	$a_{c}$
Coefficients of the complex parameters of the beam vibration	$c_{1}$ $\;\mathrm{to}\;c_{8}$
System clock frequency	$f_{clk}$
Vertical contact stiffnesses	$k^{*}$
Lateral contact stiffnesses	$k_{Lat}^{*}$
The cone radius of the probe tip	$r_{tip}$
Position of Wheatstone bridge	$u_{B}$
Junction depth	$z_{j}$
Vertical contact damping	$\gamma^{*}$
Lateral contact damping	$\gamma_{Lat}^{*}$
Mobility of the majority carriers	$\mu_{maj}$
Piezoresistive coefficient $\pi_{44}$	$\pi_{44}$
Piezoresistive coefficient	$\pi_{44}$
Density	$\rho_{m}$
Sheet resistance	$\rho_{sq}$
Uniform normal stress	$\sigma_{top,0}$
Phase delay	$\varphi_{delay}$
Sensor phase	$\varphi_{S}$
Phase shift between parasitic signal and excitation signal	$\varphi_{C}$
Resonance angular frequency	$\omega_{res}$
Phase increment value	∆θ
Cantilever thickness	*b*
Activation energy of dopant diffusion	*E*_a_
Height of diamond probe tip	*h*_tip,Dia_
Height of silicon probe tip	*h*_tip,Si_
Moment of inertia of cantilever	*I*
Proportionality factor	*k*
Boltzmann constant	*k*_B_
Mass of diamond probe tip	*m*_tip,Dia_
Mass of diamond probe tip	*m*_tip,Dia_
Mass of silicon probe tip	*m*_tip,Si_
Wave number	*q*
Radius of diamond probe tip	*r*_tip,Dia_
Radius of diamond probe tip	*r*_tip,Dia_
Cantilever width	*w*
Opening angle of diamond probe tip	*α*_tip,Dia_
Opening angle of silicon probe tip	*α*_tip,Si_
Loss factor	ζ
External damping by the surrounding air	*η*
Sheet conductance	*σ_sq_*
Angular frequency	*ω*
Net ionized doping concentration	$N\left(z\right)$
Diffusion temperature	T
Actuation frequency	f
Poisson’s ratio	ν

## Appendix A

Piezoresistivity describes the property of a resistor to transduce mechanical stress/strain into a resistivity change. Consider a homogenous resistor in the shape of a cuboid, as shown in Figure A1.

The electrical resistance ($R_{0}$) measured between its ends is

(A1) $$R_{0}=\rho_{e,0}\frac{L_{0}}{w_{0}b_{0}},$$

where $\rho_{e,0}$ is the electrical resistivity, $L_{0}$, $w_{0}$, and $b_{0}$ are the dimensions of the resistor in the *x*, *y*, and *z* directions, respectively. The resistivity of silicon depends on the dopant type, the doping concentration, and the temperature [77]. When the resistor is subjected to a force *F* in *x*-direction (*y*-direction), according to Hooke’s law [28], the resistor will be stretched, which will cause it to contract laterally in *y-* and *z*-directions (*x*- and *z*-directions). Therefore, the resistor will deform in all directions:

(A2) $$L=L_{0}\left(1+\varepsilon_{x}\right),$$

(A3) $$w=w_{0}\left(1+\varepsilon_{y}\right),$$

(A4) $$b=b_{0}\left(1+\varepsilon_{z}\right),$$

where L, w, and *b* are the dimensions of the deformed resistor, and $\varepsilon_{x}$, $\varepsilon_{y}$, and $\varepsilon_{z}$ are the strains in corresponding directions.

If the force acts in the *x*-direction, shown in Figure A1a, a longitudinal stress is generated along the *x*-direction

(A5) $$\sigma_{l}\approx \frac{F}{b_{0}w_{0}},$$

causing an increase in longitudinal strain

(A6) $$\varepsilon_{l}=\varepsilon_{x}=\frac{\sigma_{l}}{E_{m}},$$

and a reduction of strain in the *y-* and *z*-directions, i.e., the lateral strain components $\varepsilon_{y}$ and $\varepsilon_{z}$, which are proportional to the longitudinal strain according to

(A7) $$\varepsilon_{y}=\varepsilon_{z}=-\nu \varepsilon_{x}=-\nu \varepsilon_{l}=-\frac{\nu \sigma_{l}}{E_{m}}.$$

The dimensionless parameter *ν* is called Poisson’s ratio and $E_{m}$ is the Young’s modulus. Thus, the resistance changes into

(A8) $$R=\rho_{e,0}\frac{L}{wb}\;\;\;\;\;\;\;\;\;\;=\rho_{e,0}\frac{L_{0}\left(1+\varepsilon_{l}\right)}{w_{0}\left(1-\nu \varepsilon_{l}\right)b_{0}\left(1-\nu \varepsilon_{l}\right)}\;\;\;\;=R_{0}\frac{1+\varepsilon_{l}}{{\left(1-\nu \varepsilon_{l}\right)}^{2}}\;\;\;\;\;\;\;\approx R_{0}\left(1+\left(1+2\nu \right)\varepsilon_{l}\right).$$

The approximation in Equation (A8) is obtained by terminating a Taylor series expansion after the linear term [34].

If a force *F* acts in the *y*-direction, the generated stress is

(A9) $$\sigma_{t}\approx \frac{F}{b_{0}L_{0}},$$

causing strain in the *y*-direction of

(A10) $$\varepsilon_{t}=\varepsilon_{y}=\frac{\sigma_{t}}{E_{m}},$$

and strain in the other two directions of

(A11) $$\varepsilon_{x}=\varepsilon_{z}=-\nu \varepsilon_{x}=-\nu \varepsilon_{t}=-\frac{\nu \sigma_{t}}{E_{m}}.$$

Now, the resistance is given by

(A12) $$R=R_{0}\frac{1-\nu \varepsilon_{t}}{\left(1+\varepsilon_{t}\right)\left(1-\nu \varepsilon_{t}\right)}=R_{0}\frac{1}{1+\varepsilon_{t}}\;\approx R_{0}\left(1-\varepsilon_{t}\right).$$

For the approximation again a Taylor series expansion is terminated after the linear term.

In addition to the volume change, the mechanical stress has also an effect of resistivity known as the piezoresistive effect. Its physical principles can be found in [43,79,80,81,82]. Mechanical stress is transduced into a resistivity change:

(A13) $$\frac{\partial \rho_{e,0}}{\rho_{e,0}}=\pi_{l}\sigma_{l}+\pi_{t}\sigma_{t},$$

where $\pi_{l}$ and $\pi_{t}$ are the longitudinal and transverse piezoresistive coefficients, respectively. They vary with crystal direction and are a simplified representation of the piezoresistive tensor. In the case of *p*-type silicon, when the crystallographic orientation of the resistor is in <110> direction, the relationships between $\pi_{l}$, $\pi_{t}$ and the fundamental piezoresistive coefficients can be simplified [43]:

(A14) $$\pi_{l}=\frac{1}{2}\left(\pi_{11}+\pi_{12}+\pi_{44}\right)\approx \frac{\pi_{44}}{2},$$

(A15) $$\pi_{t}=\frac{1}{2}\left(\pi_{11}+\pi_{12}-\pi_{44}\right)\approx -\frac{\pi_{44}}{2}.$$

In general, the piezoresistive coefficients $\pi_{ij}\left(i,j\in \left\{1,2,4\right\}\right)$ can be modeled by the piezoresistance factor $P_{R}$ and reference values $\pi_{ij}^{ref}\left(i,j\in \left\{1,2,4\right\}\right)$ of the piezoresistive coefficients for lightly doped silicon (10^16^ cm^−3^) at 300 K [43,83]:

(A16) $$\pi_{ij}=P_{R}\left(N,T\right)\pi_{ij}^{ref}.$$

The reference piezoresistive coefficients of silicon are $\pi_{11}^{ref}={0.066\;\mathrm{GPa}}^{-1}$, $\pi_{12}^{ref}={-0.011\;\mathrm{GPa}}^{-1},$ and $\pi_{44}^{ref}={1.381\;\mathrm{GPa}}^{-1}$ [84].

There are several models available for the piezoresistance factor, like Kanda’s model [83], Harley’s model [85], and Richter’s model [86]. In this study, a modified Richter’s model presented by Doll et al. [43] is used for calculating $P_{R}$:

(A17) $$P_{R}=\frac{T_{n}^{-\vartheta }}{1+{\left(\frac{N}{N_{b}}\right)}^{\varphi }T_{n}^{-\chi }+{\left(\frac{N}{N_{c}}\right)}^{\psi }T_{n}^{-\xi }},$$

where $T_{n}=T(\in K)/300\;K$ is the normalized temperature (i.e., $T_{n}=1$ if operated under normal conditions of room temperature) and N is the doping concentration (in cm^−3^). The coefficients *ϑ*, $N_{b}$, $N_{c}$, *λ*, *χ*, *ψ*, and *ξ* are presented in Table A1.

**Table A1:** Parameters of the modified Richter model to calculate the piezoresistance factor $P_{R}$ as a function of dopant concentration and temperature.

Parameter	Modified Richter Model [41]
ϑ (−)	0.95
$N_{b}$ (cm^−3^)	4.9 × 10^19^
$N_{c}$ (cm^−3^)	2.6 × 10^20^
*λ* (−)	0.39
*χ* (−)	1.35
*ψ* (−)	0.94
*ξ* (−)	4.55

The relative resistance change is caused by both the dimensional change of the resistor (Equation (A12)) and resistivity change (Equation (A13)) [34]:

(A18) $$\frac{∆R}{R}=\left(1+2\nu \right)\varepsilon_{l}-\varepsilon_{t}+\pi_{l}\sigma_{l}+\pi_{t}\sigma_{t},$$

For a crystallographic orientation of the silicon resistor in <110> direction we have $E_{m,110}$ = 169 GPa and the corresponding Poisson ratio *ν* = 0.36 [87]. In the case of *p*-type silicon then the contribution by the geometry change compared to the contribution by the resistivity change is negligible [34]:

(A19) $$1<1+2\nu \ll 42<\frac{P_{R}\pi_{44}^{ref}E_{m,110}}{2}<109.$$

Correspondingly, we obtain finally:

(A20) $$\frac{∆R}{R}\approx \frac{\pi_{44}}{2}\sigma_{l}-\frac{\pi_{44}}{2}\sigma_{t}=\frac{\pi_{44}}{2}E_{m,110}\varepsilon_{l}-\frac{\pi_{44}}{2}E_{m,110}\varepsilon_{t}.$$

With the cantilever axis oriented in <110> direction, a full Wheatstone bridge configuration (see Figure A2) can be designed for converting the deflection of the cantilever into an electrical signal with low noise [88,89,90].

## Appendix B

The probe tips of the PCMs in Figure 2 are positioned at the bottom side of the cantilever and their contact point to a sample surface is typically located above the underside of the support frame. Therefore, the sensors can be mounted inclined to the sample surface, as shown in Figure A3. If the force *F* is applied to the contact point of the probe tip, this point is deflected by *δ* in the direction of the force. For small forces, deflection is proportional to the applied force. The shape of the sensor’s whole cantilever deflection can be calculated by the integration method [36] from the boundary conditions at both ends as well as the transition conditions of the acting forces and moments. For this purpose, the *uv*-coordinate system is used, which is rotated with respect to the *xz*-coordinate system by the angle of attack *θ*.

Both coordinate systems are connected via the following transformation equations:

(A21) $$\left\{\begin{array}{l} u=xcos\theta -zsin\theta ,\\ v=xsin\theta +zcos\theta , \end{array}\right.$$

(A22) $$\left\{\begin{array}{l} x=ucos\theta +vsin\theta ,\\ z=-usin\theta +vcos\theta . \end{array}\right.$$

Due to the fixed clamping at *u* = 0 and the free end at $u_{2}$ = $L_{1}$ + $L_{2}$, the following boundary conditions apply:

(A23) $$\left\{\begin{array}{l} v\left(0\right)=0,\\ \frac{\partial v}{\partial u}\left(0\right)=0, \end{array}\right.$$

(A24) $$\left\{\begin{array}{l} M\left(u_{2}\right)=E_{m}I\frac{\partial^{2}v}{\partial u^{2}}\left(u_{2}\right)=0,\\ Q_{m}\left(u_{2}\right)=E_{m}I\frac{\partial^{3}v}{\partial u^{3}}(u_{2})=0, \end{array}\right.$$

where *v* is the deflection of the neutral fiber of the beam, *I* is the area moment of inertia given by:

(A25) $$I=\frac{b^{3}}{12}\times w,$$

M is the bending moment and $Q_{m}$ is the shear force, for which the applied force *F* creates transition conditions between segment 1 and 2 of the cantilever, i.e., at the position of the probe tip $u_{1}$ = $L_{1}$:

(A26) $$\left\{\begin{array}{c} Q_{m}\left(u_{1}^{+}\right)=Q_{m}\left(u_{1}^{-}\right)-Fcos\theta ,\\ M\left(u_{1}^{+}\right)=M\left(u_{1}^{-}\right)-FDsin\theta . \end{array}\right.$$

Here, $Q_{m}\left(u_{1}^{+}\right)$ and $Q_{m}(u_{1}^{-})$ (or $M\left(u_{1}^{+}\right)$ and $M\left(u_{1}^{-}\right)$) are the right-hand and left-hand limit values of $Q_{m}\left(u_{1}\right)$ (or ${M(u}_{1})$). *D = h + b*/2 is the distance between the contact point of the probe tip and the neutral fiber of the beam.

The position of the contact point of the probe tip is determined by the deflection and slope of the neutral fiber (see Figure A4 and Figure A5):

(A27) $$Q_{m}\left(u\right)=\left\{\begin{array}{c} Fcos\theta :0\leq u\leq L_{1},\\ 0:L_{1}\leq u\leq L_{1}+L_{2}, \end{array}\right.$$

(A28) $$M\left(u\right)=\left\{\begin{array}{c} F\left(cos\theta \left[u-L_{1}\right]\right)+Dsin\theta :0\leq u\leq L_{1},\\ 0:L_{1}<u\leq {L_{1}+L}_{2}, \end{array}\right.$$

(A29) $$\frac{\partial v}{\partial u}\left(u\right)=\left\{\begin{array}{c} v_{1}^{\prime }\left(u\right)=\frac{6F}{E_{m}wb^{3}}\left(cos\theta \left[-u^{2}+2L_{1}u\right]-2Dsin\theta u\right):0\leq u\leq L_{1},\\ v_{2}^{\prime }\left(u\right)=\frac{6F}{E_{m}wb^{3}}\left(cos\theta L_{1}^{2}-2Dsin\theta L_{1}\right):L_{1}<u\leq {L_{1}+L}_{2}, \end{array}\right.$$

(A30) $$v\left(u\right)=\left\{\begin{array}{c} v_{1}\left(u\right)=\frac{2F}{E_{m}wb^{3}}\left(cos\theta \left[-u^{3}+3L_{1}u^{2}\right]-3Dsin\theta u^{2}\right):0\leq u\leq L_{1},\\ v_{2}\left(u\right)=\frac{2F}{E_{m}wb^{3}}(cos\theta \left[3L_{1}^{2}u-L_{1}^{3}\right]-3Dsin\theta [2L_{1}u-{LL}_{1}^{2}]):L_{1}<u\leq {L_{1}+L}_{2} \end{array}\right.$$

Figure A5 shows an enlarged view of the relevant detail of the cantilever at the position of the tip. The angle *β* describes the slope of the deflection *v* of the neutral fiber of the cantilever with *u* at the position of the probe tip.

It is given by

(A31) $$\beta =arctan\frac{\partial v}{\partial u}\left(u_{1}\right),$$

For the tip coordinates we obtain with trigonometric addition theorems [91]:

(A32) $$u_{tip}=u_{1}+Dsin\beta ,\;\;\;\;\;\;\;\;=u_{1}+Dsin[arctan\frac{\partial v}{\partial u}\left(u_{1}\right)],\;\;\;\;\;\;\;\;\;\;\;=u_{1}+\frac{\partial v}{\partial u}\left(u_{1}\right)\frac{D}{\sqrt{1+{\left(\frac{\partial v}{\partial u}\left(u_{1}\right)\right)}^{2}}},$$

(A33) $$v_{tip}=v\left(u_{1}\right)-Dcos\beta ,\;\;\;\;\;\;\;\;=v\left(u_{1}\right)-Dcos[arctan\frac{\partial v}{\partial u}\left(u_{1}\right)],\;\;\;\;\;\;\;\;=v\left(u_{1}\right)-\frac{D}{\sqrt{1+{\left(\frac{\partial v}{\partial u}\left(u_{1}\right)\right)}^{2}}}.$$

Without applied force, the tip position is at:

(A34) $$u_{tip,0}=u_{1}\wedge v_{tip,0}=-D.$$

and its deflection in *z* direction is given by the transformation:

(A35) $$\delta =z_{tip}-z_{tip,0}=-\left(u_{tip}-u_{tip,0}\right)sin\theta +\left(v_{tip}-v_{tip,0}\right)cos\theta$$

Inserting Equations (A29), (A30), (A32), (A33)–(A35), and neglecting nonlinear effects, leads to the spring constant *k_c_* of the inclined beam, with:

(A36) $$k_{c}=\frac{F}{\delta }\approx \frac{E_{m}wb^{3}}{2}\frac{1}{2{L_{1}^{3}cos}^{2}\theta +3DL_{1}(2D{sin}^{2}\theta -L_{1}sin2\theta )}$$

With an angle of attack of 0° (or 15°), the stiffness of the PCM (Table 1) is 8.7 Nm^−1^ (or 9.5 Nm^−1^).

In the case of bending deformation caused by a vertical force acting on the beam, the normal stress along the cantilever axis is given via the relationship [92,93]:

(A37) $$\sigma =\frac{vM}{I}$$

For a beam of constant width (as the PCM of Table 1), the magnitude of this value for small angles of attack is maximum on the top or bottom surface of the beam (at *v* = ±*b*/2) and at its clamping (at *u* = 0). The value of the normal stress on the top surface is derived from Equations (A25) and (A28) as:

(A38) $$\sigma_{top,0}=\frac{6F}{wb^{2}}\left(Dsin\theta -L_{1}cos\theta \right)$$

Due to fabrication issues the top-surface position of maximum stress is therefore selected for measuring the applied force or the induced tip deflection using a piezoresistive Wheatstone bridge (see Appendix A):

(A39) $$F=\frac{wb^{2}\sigma_{top,0}}{6(Dsin\theta -L_{1}cos\theta )}$$

(A40) $$\delta =\frac{F}{k_{c}}$$

## Appendix C

Compared to the boundary conditions of the static deflection given by Equations (A23) and (A24), a boundary condition must be extended here to account for the case of dynamic excitation [47,94,95,96]:

(A41) $$\left\{\begin{array}{c} v\left(0,t\right)=v_{0}cos\omega t,\\ \frac{\partial v}{\partial u}\left(0,t\right)=0,\\ \frac{\partial^{2}v}{\partial u^{2}}\left(u_{2},t\right)=0,\\ \frac{\partial^{3}v}{\partial u^{3}}\left(u_{2},t\right)=0, \end{array}\right.$$

Position of the neutral fiber, its slope, bending moment and transverse force are obtained under these conditions as:

(A42) $$\left\{\begin{array}{c} v\left(L_{0}^{+},t\right)=v\left(L_{0}^{-},t\right),\\ \frac{\partial v}{\partial u}\left(L_{0}^{+},t\right)=\frac{\partial v}{\partial u}\left(L_{0}^{-},t\right), \end{array}\right.$$

(A43) $$\left\{\begin{array}{c} E_{m}I\frac{\partial^{2}v}{\partial u^{2}}\left(L_{0}^{+},t\right)=E_{m}I\frac{\partial^{2}v}{\partial u^{2}}\left(L_{0}^{-},t\right),\\ E_{m}I\frac{\partial^{3}v}{\partial u^{3}}\left(L_{0}^{+},t\right)=E_{m}I\frac{\partial^{3}v}{\partial u^{3}}\left(L_{0}^{-},t\right), \end{array}\right.$$

(A44) $$\left\{\begin{array}{c} v\left(u_{1}^{+},t\right)=v\left(u_{1}^{-},t\right),\\ \frac{\partial v}{\partial u}\left(u_{1}^{+},t\right)=\frac{\partial v}{\partial u}\left(u_{1}^{-},t\right). \end{array}\right.$$

In addition, the acceleration of the probe tip causes an inertial force due to its mass $m_{tip}$, with the following boundary conditions for bending moment and transverse force apply [65,94,95,96]:

(A45) $$E_{m}I\frac{\partial^{2}v}{\partial u^{2}}\left(u_{1}^{+},t\right)=E_{m}I\frac{\partial^{2}v}{\partial u^{2}}\left(u_{1}^{-},t\right)-\kappa^{2}m_{tip}\omega^{2}\frac{\partial v}{\partial u}\left(u_{1}^{-},t\right),$$

(A46) $$E_{m}I\frac{\partial^{3}v}{\partial u^{3}}\left(u_{1}^{+},t\right)=E_{m}I\frac{\partial^{3}v}{\partial u^{3}}\left(u_{1}^{-},t\right)-m_{tip}\omega^{2}v\left(u_{1}^{-},t\right),$$

where *κ* is the distance between the center of gravity of the probe tip and the neutral fiber of the beam.

For a conical probe tip, we have:

(A47) $$\kappa =\frac{h}{3}+\frac{b}{2}.$$

Furthermore, its mass is determined by:

(A48) $$m_{tip}=\frac{1}{3}\rho_{m}h^{3}\pi {tan}^{2}\frac{\alpha }{2},$$

where *α* is the opening angle of the tip cone.

## Appendix D

To account for viscous properties of tip and sample, the indentation modulus $M_{m}$ and the shear modulus $G_{m}$ [61,62], which each describe purely elastic behavior, are expanded by a loss factor *ζ* to form the complex indentation modulus $\underline{M_{m}}$ and the complex shear modulus $\underline{G_{m}}$:

(A49) $$\underline{M_{m}}=\left(1+i\zeta \right)M_{m},$$

(A50) $$\underline{G_{m}}=\left(1+i\zeta \right)G_{m},$$

The loss factors of different materials can be found in [97].

The complex reduced Young’s modulus $\underline{E_{m}^{*}}$ and the complex reduced shear modulus $\underline{G_{m}^{*}}$ are then given by:

(A51) $$\frac{1}{\underline{E_{m}^{*}}}=\frac{1}{\underline{M_{m,s}}}+\frac{1}{\underline{M_{m,tip}}}=:\frac{1}{E_{m}^{{*}^{\prime }}+{iE}_{m}^{{*}^{\prime \prime }}},$$

(A52) $$\frac{1}{\underline{G_{m}^{*}}}=\frac{2-v_{s}}{\underline{G_{m,s}}}+\frac{2-v_{tip}}{\underline{G_{m,tip}}}=:\frac{1}{G_{m}^{{*}^{\prime }}+{iG}_{m}^{{*}^{\prime \prime }}},$$

where $\underline{M_{m,tip}}$, $\underline{G_{m,tip}}$ and $v_{tip}$ are the complex indentation modulus, the complex shear modulus and the Poisson’s ratio of the material of the probe tip; $\underline{M_{m,s}}$, $\underline{G_{m,s}}$ and $v_{s}$ are the values of the sample material.

## Appendix E

When force is applied to the tip being in contact to a sample the boundary conditions for bending moment and transverse force at the position *u* = *u*_1_ close to the free end of the cantilever change to:

(A53) $$\begin{array}{l} E_{m}I\frac{\partial^{2}v}{\partial u^{2}}\left(u_{1}^{+},t\right)=E_{m}I\frac{\partial^{2}v}{\partial u^{2}}\left(u_{1}^{-},t\right)\\ +\left(D^{2}\left[\left(k^{*}+i\omega r^{*}\right){sin}^{2}\theta +\left(k_{Lat}^{*}+i\omega r_{Lat}^{*}\right){cos}^{2}\theta \right]-\kappa^{2}m_{tip}\omega^{2}\right)\frac{\partial v}{\partial u}\left(u_{1}^{-},t\right)\\ +Dsin\theta cos\theta \left[k_{Lat}^{*}+i\omega r_{Lat}^{*}-k^{*}-i\omega r^{*}\right]v\left(u_{1}^{-},t\right), \end{array}$$

(A54) $$\begin{array}{l} E_{m}I\frac{\partial^{3}v}{\partial u^{3}}\left(u_{1}^{+},t\right)=E_{m}I\frac{\partial^{3}v}{\partial u^{3}}\left(u_{1}^{-},t\right)\\ -Dsin\theta cos\theta \left[k_{Lat}^{*}+i\omega r_{Lat}^{*}-k^{*}-i\omega r^{*}\right]\frac{\partial v}{\partial u}\left(u_{1}^{-},t\right)\\ -\left(\left[k^{*}+i\omega r^{*}\right]{cos}^{2}\theta +\left[k_{Lat}^{*}+i\omega r_{Lat}^{*}\right]{sin}^{2}\theta -m_{tip}\omega^{2}\right)v\left(u_{1}^{-},t\right), \end{array}$$

$v\left(u_{1}^{-},t\right)$ and $v\left(u_{1}^{-},t\right)$ (as well as their derivatives) denote the right-hand and left-hand limit values of $v\left(u_{1}\right)$ (or its derivatives). The boundary conditions at the cantilever clamping (*u* = 0) from Equation (A41) to (A44) remain unchanged.

## Appendix F

A new PCM with PEA has been designed in this study (see Figure 2b). Using PEA, the polarity of the induced strain/stress is determined by the sign of the applied voltage/electric field. In the present design, the electrodes of two actuators are configured that polarity of the applied electric field is opposite. Thereby, the second bending mode can be effectively stimulated with two actuators (see Figure 11). In this mode the largest effect of contact interaction between tip and sample can be expected (see Figure 12 and Table 3).

## Appendix G

Finite element analysis (FEM) of the piezoelectric cantilever microprobe is performed using COMSOL Multiphysics 5.4. To save computational time the complex structure of the microprobe is simplified, e.g., by assuming a square frame for the Wheatstone bridge. A hexahedral mesh is taken for the beam and tetrahedral mesh for the tip, with a smooth transition between both and a finer mesh at the contacting boundary from the tip to a sample (whose boundary is at least twice finer meshed than the tip boundary). The total number of elements is 25,270. A rotated coordinate system (by 45° about the *z*-axis ([001] direction towards *x* in [110] direction) is employed corresponding to the cantilever axis orientation. The tip-sample contact pair is modeled as a spring, for the base of the cantilever a fixed constraint is assumed. Ideal inverse piezoelectric actuation in *z*-direction is assumed in the AlN sheet. Additional deformation of the cantilever due to thermal expansion caused by Joule heating of the resistive elements of the Wheatstone bridge is considered.

The process steps of FEA are shown in Figure A7, and the properties and dimensions of the used materials in Table A2. In the first step, “Heat Transfer in Solid” is modeled by activating the modules “Piezoresistive Effect”, “Temperature Coupling,” and “Thermal Expansion”, including the modules “Solid Mechanics” and “Electric Currents” to simulate the piezoresistive effect. Using these results as initial values, in the second step, the total displacement of the tip is modeled using the modules “Solid Mechanics” and “Electrostatics” as well as the module “Piezoelectric Effect”. Then, the condition tip in contact with the sample is modeled for analyzing the deformation of the sample and the deflection of the cantilever using the module “Solid Mechanics”. Here, the previously calculated results were automatically adopted, i.e., all effects are coupled. Finally, harmonic vibrations of the cantilever are modeled by supplying the piezoelectric actuator with an AC voltage.

**Table A2:** Properties of the components of the piezoelectric PCM used in the numerical simulations by FEA.

Material	Gold	SiO_2_	AlN	Silicon	Diamond
Layer thickness	0.5 μm	0.5 μm	1 μm	50 μm	- *
Young’s modulus	70 GPa	70 GPa	330 GPa	170 GPa	1050
Loss factor	0.001	0.001	0.001	- *	0.001

* Values not needed for simulation.

## Appendix H

**Table A3:** Process parameters used for boron dopant (PBF2.2DS) and phosphorus dopant (P509) diffusion.

Parameter	Boron Dopant (PBF2.2DS)	Phosphorus Dopant (P509)
Spin on	500 rpm, 2 s 4000 rpm, 30 s	500 rpm, 2 s 4000 rpm, 30 s
Bake	150 °C, 1 min	Not required
Diffusion	*T* *, 30 min 4 L/min N_2_ + 0.5 L/min O_2_ (dry)	*T* *, 30 min 4 L/min N_2_ + 0.5 L/min O_2_ (dry)

* *T* = 900 °C; 1000 °C; 1100 °C, 1200 °C.

**Table A4:** The values of the parameters from Gaussian normal distribution fitting (Equation (23)) of the electrically active dopant concentration profiles of boron (PBF2.2DS, Filmtronics, Butler, PA, USA) in *n*-type silicon (100) at different processing temperatures (Figure 13).

Temperature	*n*	$a_{1}$	$b_{1}$	$c_{1}$	$a_{2}$	$b_{2}$	$c_{2}$	$a_{3}$	$b_{3}$	$c_{3}$
900	2	1.34 × 10^34^	−22.03	3.867	−7.87 × 10^19^	0.0977	0.1112	-	-	-
1000	3	4.33 × 10^19^	0.1057	0.1166	0	147.7	23.71	8.15 × 10^18^	0.2402	0.07112
1100	3	0	0.5222	0.003066	9.95 × 10^19^	0.2359	0.8629	2.89 × 10^19^	1.199	0.6026
1200	2	7.55 × 10^19^	0.1554	0.9457	4.51 × 10^19^	1.442	1.027	-	-	-

**Table A5:** Sheet resistance (Ω/sq) of boron dopant (PBF2.2DS, Filmtronics, Butler, PA, USA) in n-type silicon (100) at different doping temperatures.

Temperature (°C)	ECV&CV	FPP	Supplier
900	401.87	403.32	132
950	- *	- *	64
1000	110.12	127.36	15
1050	- *	- *	8
1100	9.25	10.39	- *
1150	- *	- *	5
1200	7.55	7.91	3.5

* There are no data taken at this temperature or provided by the supplier.

**Table A6:** Sheet resistance (Ω/sq) of phosphorus (P509, Filmtronics, Butler, PA, USA) in n-type silicon (100) at different doping temperatures.

**Temperature (** **°C)**	**ECV&CV**	**PFF**	**Supplier**
900	208.61	142.72	23
950	- *	- *	7
1000	13.11	15.75	5.5
1050	- *	- *	4
1100	4.15	4.88	- *
1150	- *	- *	2.8
1200	2.6	3.24	2

* There are no data taken at this temperature or provided by the supplier.
